# Expanding the genetic spectrum of inherited peripheral neuropathies in complex neurogenetic disorders using whole-exome sequencing

**DOI:** 10.1007/s10048-026-00952-w

**Published:** 2026-10-29

**Authors:** Paria Najarzadeh Torbati, Reza Boostani, Mojtaba Safi, Najmeh Ahangari, Mohammad Doosti, Hasan Badpar, Mehran Beiraghi Toosi, Ehsan Ghayoor Karimiani, Barbara Vona

**Affiliations:** 1 Department of Medical Genetics, Next Generation Genetic Polyclinic, Mashhad, Iran; 2 Department of Biology, Mashhad Branch, Islamic Azad University, Mashhad, Iran; 3 Department of Neurology, Mashhad University of Medical Sciences, Mashhad, Iran; 4 Rare Pediatric Neurological Diseases Research Center, Mashhad University of Medical Sciences, Mashhad, Iran; 5 Centre for Neuromuscular Diseases, UCL Queen Square Institute of Neurology, London, WC1N 3BG UK; 6 Institute for Auditory Neuroscience and Inner Ear Lab, University Medical Center Göttingen, Göttingen, Germany; 7 Auditory Neuroscience and Optogenetics Laboratory, German Primate Center, Göttingen, Germany; 8 Collaborative Research Center 1690 (CRC 1690), University of Göttingen, Göttingen, Germany; 9 Department of Obstetrics and Gynecology, Brigham and Women’s Hospital, Harvard Medical School, Boston, MA USA; 10 Program in Medical and Population Genetics, Broad Institute of MIT and Harvard, Cambridge, MA USA

**Keywords:** Inherited peripheral neuropathy, Hereditary sensory and motor neuropathy, Peripheral nervous system diseases, Whole exome sequencing

## Abstract

Inherited peripheral neuropathies (IPNs) represent a clinically and genetically heterogeneous group of disorders affecting sensory, motor, autonomic functions, and can also present as a part of complex neurogenetic syndromes. Charcot-Marie-Tooth disease (CMT) is the most prevalent type of IPN. Despite advances in genetic testing, the diagnostic process remains challenging due to genetic heterogeneity and overlapping clinical phenotypes. We performed a comprehensive genetic evaluation of 126 patients presenting with symptoms of IPN. Nineteen patients with a typical CMT phenotype were initially screened for PMP22 copy number variations using Multiplex Ligation-dependent Probe Amplification (MLPA). Whole-exome sequencing (WES) was subsequently performed in 107 patients. Of these, 72 (63.7%) were classified as pathogenic or likely pathogenic, including 36 novel variants (50.0%), yielding an overall WES diagnostic rate of 62.6% (67/107). CMT-spectrum disorders identified by MLPA (n = 19 patients) and WES (n = 33 patients) accounted for 41.3% (52/126) of the study cohort. Additional diagnoses included hereditary sensory neuropathy 3.97% (5/126), distal hereditary motor neuropathy 4.76% (6/126), spastic paraplegia 3.97% (5/126), spinocerebellar ataxia 5.56% (7/126), spastic ataxia 8.73% (11/126), mitochondrial disease 7.94% (10/126), and other complex neurogenetic disorders 23.81% (30/126). Our findings highlight the utility of genomic sequencing approaches for resolving clinically heterogeneous neuropathy presentations and demonstrate the value of WES in a highly consanguineous Iranian cohort. These results support the role of WES in advancing precision medicine approaches for inherited neuropathies.

**Supplementary Information:**

The online version contains supplementary material available at https://doi.org/10.1007/s10048-026-00952-w.

## Introduction

Inherited peripheral neuropathies (IPNs) are a heterogeneous group of neurodegenerative disorders characterized by dysfunction of sensory, motor, or autonomic nerves. These disorders show symptoms that can vary across different developmental stages [1, 2]. Among IPNs, CMT, HSN, and dHMN represent major clinical categories in which peripheral nerve dysfunction is the predominant driver of the phenotype. CMT is characterized by a combination of motor and sensory impairment, typically presenting with distal muscle weakness, sensory loss, foot deformities, and reduced reflexes [3]. HSN primarily affects sensory and autonomic neurons, leading to sensory loss, impaired pain perception, recurrent injuries, and variable autonomic dysfunction. dHMN affects motor neurons and is characterized by progressive distal muscle weakness and atrophy with minimal or absent sensory involvement [4]. Despite these clinical distinctions, substantial phenotypic overlap and genetic heterogeneity exist among these disorders, often complicating clinical classification and diagnosis [5]. In this study, we use the term “pure IPNs” to describe disorders in which peripheral neuropathy represents the predominant or sole clinical manifestation. This category includes CMT, HSN, and dHMN. In addition, IPNs may appear as a prominent clinical feature of complex neurogenetic disorders, such as hereditary spastic paraplegias (HSP) and spinocerebellar ataxias (SCA), mitochondrial or neurodevelopmental disorders, further complicating the diagnostic process [4, 5].

To date, variants in over 130 genes have been associated with pure IPNs and related disorders, highlighting the genetic heterogeneity of these disorders [3, 6]. Whole-exome sequencing (WES) has proven effective in diagnosing many of these cases [7]. The prevalence of IPNs is estimated to be less than 1% in the general population [8]. Studies from African and Middle Eastern populations have reported lower prevalence rates of 0.08–0.25% ( 0.8–2.5 per 1,000 individuals) [8]. Among inherited forms, CMT is the most common, affecting approximately 40 to 82 individuals per 100,000 [9]. The underlying variants affect genes encoding proteins involved in myelin structure, axonal transport, and mitochondrial metabolism, leading to different CMT subtypes. These subtypes are classified according to the pathological and genetic findings, including demyelinating (CMT1, CMT3, and CMT4), axonal (CMT2), and mixed or intermediate forms (CMTX and dominant intermediate CMT) [10]. Identifying a specific gene involved in disease is important for targeted therapeutic approaches. In this regard, nerve conduction studies, clinical severity, symptom profile, age of onset, and additional neurological signs are all valuable for subtyping the disease and genetic evaluation [10, 11]. Electrophysiological assessments characterize demyelinating, axonal, and intermediate forms. Demyelinating forms are characterized by reduced nerve conduction velocities, whereas axonal forms typically show reduced amplitudes of sensory and motor nerve action potentials. Intermediate forms display both demyelination and axonal loss, with disease progression over months to years [12].

Distinguishing between acquired and inherited forms, as well as between acute or chronic, and axonal or demyelinating subtypes, is vital for clinical diagnosis and management [13]. Guidelines have been reported to support these differentiations [14]. Additionally, the World Health Organization (WHO) has developed a protocol aimed at studying major neurological disorders in developing countries. The first stage of this protocol includes a clinical survey and neurological examinations conducted under the supervision of a neurologist. Participants undergo further evaluation to determine the specific type of neurological disorder, including epilepsy and peripheral neuropathy. In Middle Eastern countries, the reported prevalence of polyneuropathy is relatively low [15]. In this cohort study conducted in northeastern Iran, we studied 126 patients with pure IPNs and complex neurogenetic disorders with prominent IPN clinical features. Patients either received a molecular diagnosis or carry candidate disease-associated variants. The diagnostic yield in complex cases can be improved through deep analysis of WES data, especially when earlier targeted genetic testing has failed. Incorporating recently discovered genes and resolving complex phenotypic presentations play an important role in achieving high diagnostic yield in patients [16].

Despite advances in NGS technologies, the genetic diagnosis of IPN remains challenging due to phenotypic overlap and genetic heterogeneity [5]. Additionally, epidemiological data from Middle Eastern populations are limited [17, 18]. This regional cohort study aimed to characterize the genetic landscape of IPNs across a broad spectrum of neurological disorders, encompassing both primary inherited neuropathies and syndromic conditions in which peripheral neuropathy represents a major clinical feature, including spastic ataxia, hereditary spastic paraplegia, mitochondrial diseases, and neurodevelopmental or neurodegenerative conditions. 

## Methods

### Study design and participant recruitment

**Fig. 1:**
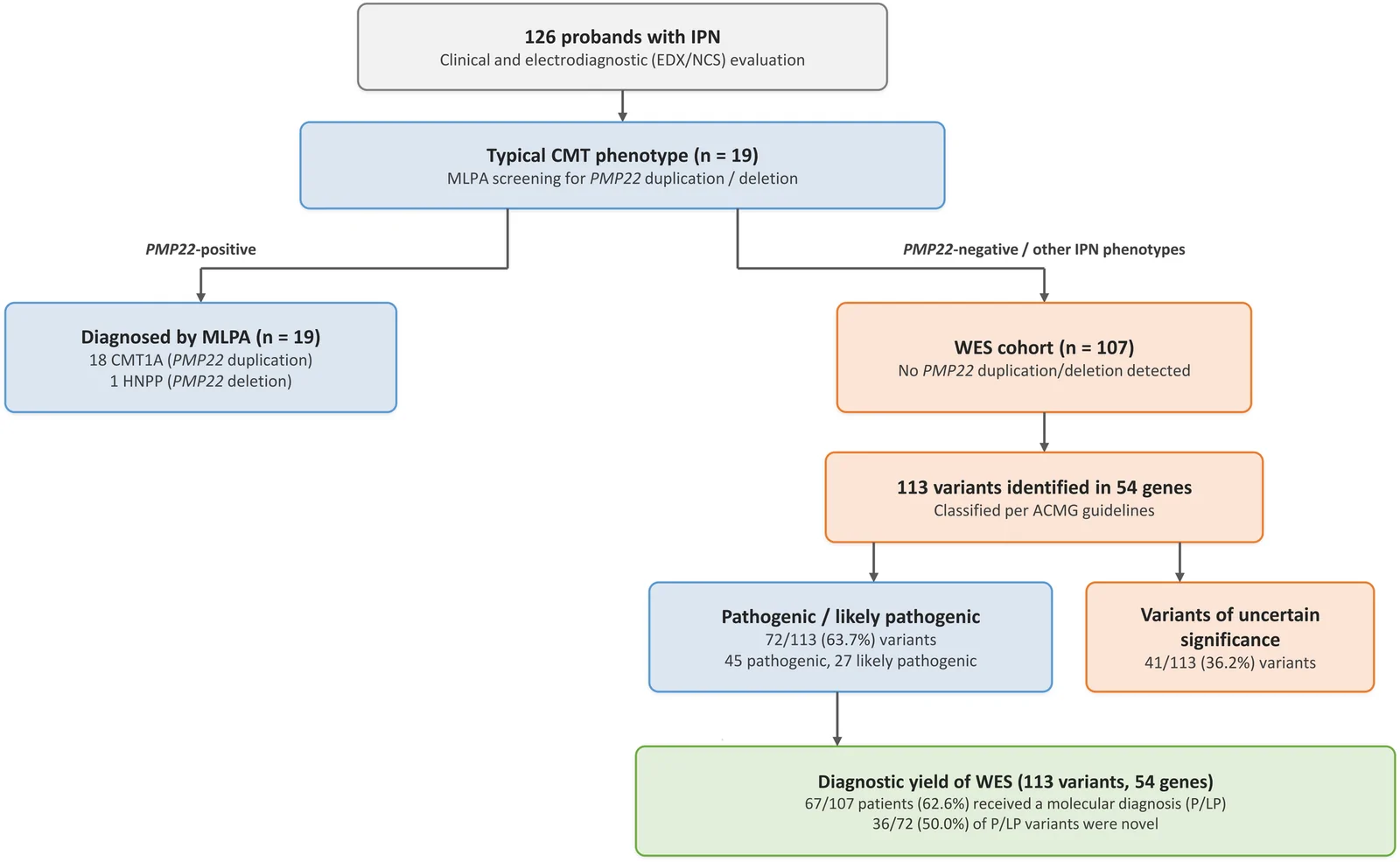
Genetic testing algorithm. Stepwise diagnostic workflow from initial clinical evaluation through *PMP22* MLPA screening and WES, with variant classification outcomes and diagnostic yield at each stage. CMT, Charcot-Marie-Tooth disease; HNPP, hereditary neuropathy with liability to pressure palsies; IPN, inherited peripheral neuropathy; P/LP, pathogenic/likely pathogenic; MLPA, multiplex ligation-dependent probe amplification; WES, whole-exome sequencing; ACMG, American College of Medical Genetics and Genomics

This study was performed with ethical approval from the Mashhad University of Medical Sciences (approval number: IR.MUMS.MEDICAL.REC.1399.704). A total of 126 patients presenting with clinical symptoms suggestive of IPNs were referred by neurologists from the Next Generation Genetic Polyclinic for genetic testing. Affected relatives were not included in this count. Patient inclusion was based on clinical presentation, the neurologists’ clinical assessment and suspicion of IPNs ; electrodiagnostic (EDX) findings were incorporated when available but were not a mandatory inclusion criterion. The stepwise genetic testing algorithm applied to the cohort is illustrated in Fig. 1. Clinical categorization was assigned retrospectively based on review of clinical records, neurological examinations, and EDX findings documented by the treating neurologists prior to genetic testing. In patients lacking EDX data, classification was based on the predominant clinical syndrome, supported by neurological examination findings. Following WES analysis, diagnoses were reviewed and refined when the genetic findings supported a broader or alternative neurogenetic diagnosis. CMT was defined by length-dependent motor and sensory neuropathy with distal weakness and sensory impairment [12]. HSP was defined by progressive lower-limb spasticity and pyramidal signs as the dominant clinical feature [19]. SCA was defined by progressive cerebellar ataxia as the predominant manifestation [20]. Spastic ataxia was diagnosed when both cerebellar ataxia and pyramidal tract dysfunction were prominent and clinically significant [21]. dHMN was characterized by motor neuropathy with minimal or absent sensory involvement [22], whereas HSN was characterized by sensory and/or autonomic dysfunction [23]. Other categories were assigned according to the leading neurological phenotype at presentation. Diagnostic classification was consistent with established clinical guidelines and OMIM disease definitions (www.omim.org). For the purposes of this study, patients were categorized as having pure IPNs when peripheral neuropathy was the predominant clinical manifestation, including CMT, HSN, and dHMN. Disorders such as HSP, SCA, mitochondrial diseases, and neurodevelopmental or neurodegenerative conditions were classified as complex neurogenetic disorders when peripheral neuropathy occurred as part of a broader neurological phenotype.

All nine categories, including CMT, dHMN, HSN, HSP, SCA, spastic ataxia, mitochondrial disease, other neuropathy syndromes, and other complex neurogenetic disorders (including pontocerebellar hypoplasia, NESCAV syndrome, amyotrophic lateral sclerosis, and other neurodevelopmental or neurodegenerative disorders), are summarized in Fig. 1.

All probands, or their legal guardians when applicable, provided written informed consent for NGS analysis and for the use of their data in subsequent publications. Phenotypic information reported in this study is based on available clinical and EDX findings and does not reflect the full spectrum of clinical manifestations.

### Genetic analysis

Genomic DNA from probands was extracted from peripheral blood. The ReliaPrep Promega kit was used for patients undergoing MLPA testing, while a standard salting-out method was applied for individuals analyzed by WES. Affected individuals with a clinical and EDX suspicion of *CMT1A* were screened using SALSA MLPA Probemix P033 kit to detect duplications (MIM 118220) or deletions (MIM 162500) of *PMP22*. Individuals with negative results and those with other forms of suspected inherited neuropathies proceeded to WES, using the SureSelect Agilent v7 exome kit for the preparation of the exome library, and sequencing was performed on an Illumina HiSeq 4000 system (Illumina, Inc., San Diego, CA, USA). WES-based copy-number variation (CNV) analysis for *PMP22* was not performed in this cohort. Raw sequencing reads were aligned to the human reference genome (GRCh38), using Burrows-Wheeler Aligner (BWA), followed by variant calling (GATK 4). SAM files were converted to BAM, sorted, and duplicate reads were removed using Picard (http://picard.sourceforge.net*).* Variant annotation was performed using ANNOVAR (v2025). Variant filtering was based on quality metrics including read depth, genotype quality, and variant quality scores. Common variants were excluded using a minor allele frequency cutoff of < 1% in public databases such as the Genome Aggregation Database (gnomAD). A variant was designated as novel if it had not been previously reported in variant databases or the peer-reviewed literature. For variant prioritization, we employed multiple in silico tools to predict pathogenicity, including SIFT, Mutation Assessor, CADD, and MutationTaster. Variants were classified according to the American College of Medical Genetics and Genomics (ACMG) guidelines [24]. Variants of Uncertain Significance (VUS) were not considered diagnostic. However, they were documented if they had a plausible link to the phenotype according to gene function and expression, inheritance pattern, and variant location. Classification was based on an integrated assessment of population frequency, computational prediction, published functional data, segregation, and previously reported clinical evidence. All reported pathogenic/likely pathogenic variants were confirmed by Sanger sequencing in the proband. Segregation analysis was performed in available family members. In cases consistent with autosomal dominant (AD) inheritance where parental samples were available, de novo occurrence of the variants was confirmed and for those families with unavailable samples, validation was limited to the proband. Comparison of diagnostic yield between phenotypic categories (pure IPN vs. complex neurogenetic disorders) was performed using Fisher’s exact test (SciPy v1.18.1, Python 3), with *p* < 0.05 considered statistically significant.

## Results

**Fig.2:**
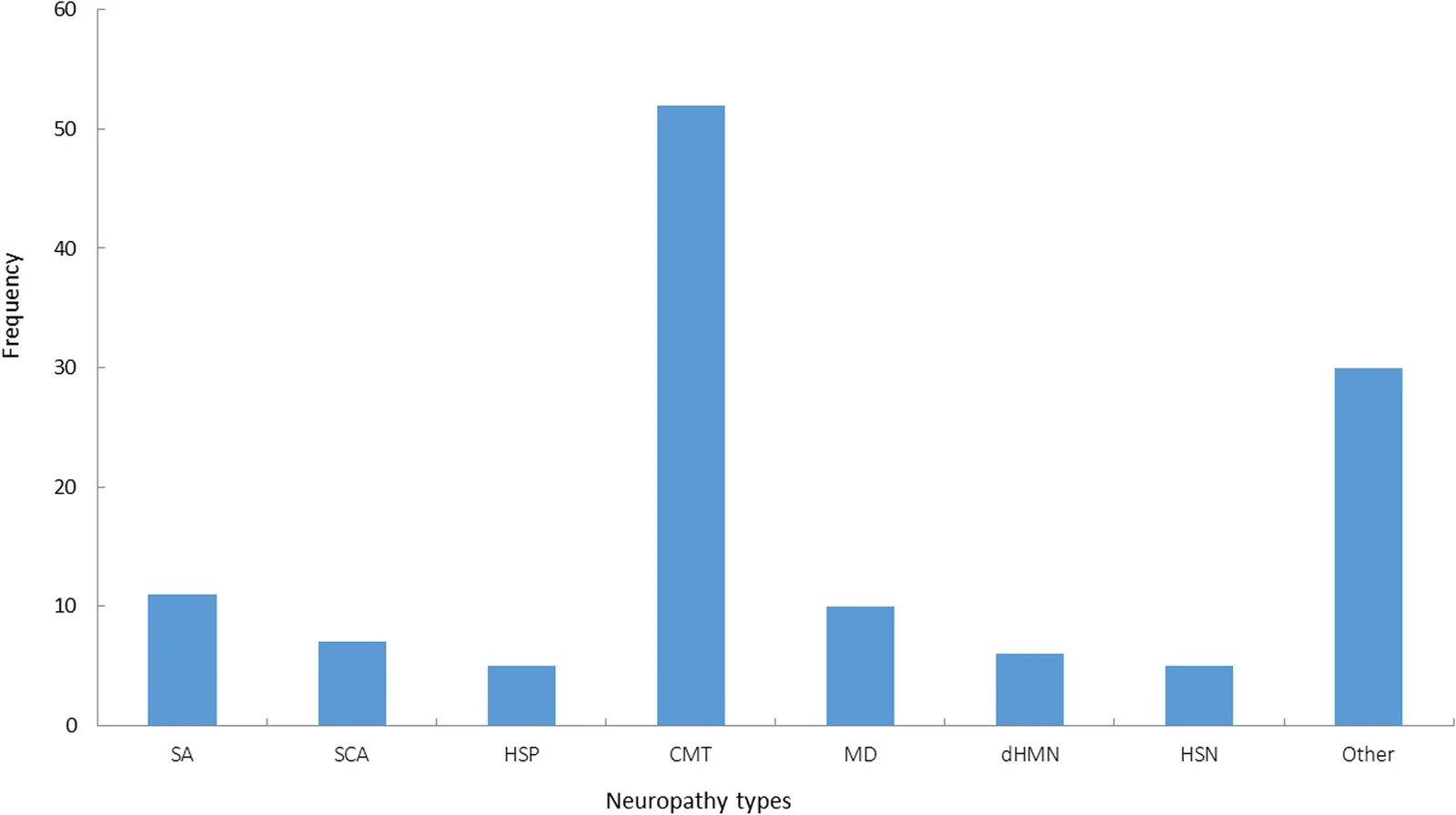
Distribution of patients according to neuropathy subtype. The figure illustrates the frequency of patients diagnosed with various inherited neurological disorders, including spastic ataxia (SA), spinocerebellar ataxia (SCA), hereditary spastic paraplegia (HSP), Charcot-Marie-Tooth disease (CMT), mitochondrial disease (MD), distal hereditary motor neuropathy (dHMN), hereditary sensory neuropathy (HSN). The other complex neurogenetic disorders and other neuropathy syndromes’ categories comprise additional rare diagnoses; the full list of associated OMIM phenotypes is provided in Supplementary Table 1

All 126 individuals included in this study were probands. The distribution of patients according to neuropathy subtype is shown in Fig. 2. Among 126 patients, 19 individuals, 13 males and 6 females (average age: 27 years, range: 4–41 years), from parents with no consanguinity were diagnosed through MLPA analysis targeting *PMP22.* Of these, 18 patients had duplications consistent with CMT type 1A (CMT1A), and one had a deletion, which causes hereditary neuropathy with liability to pressure palsies (HNPP). We identified 113 variants in 54 genes in the remaining 107 unrelated patients. Of the 107 probands, 52 were male and 55 were female. A total of 83 patients came from consanguineous families. The inheritance pattern among the 107 probands analyzed by WES was classified as follows: 81 probands (75.7%) showed autosomal recessive (AR) inheritance, and 25 cases (23.4%) showed AD inheritance, and one proband (P91) presented with two distinct molecular analysis showing both AR and AD inheritance. No X-linked cases were identified. Figure 1 illustrates the diagnostic algorithm, with WES establishing a molecular diagnosis in 67 of 107 (pathogenic or likely pathogenic variants) patients (62.6%).

Available information of 107 enrolled probands studied by WES is provided in Supplementary Table 1. This includes age of onset, parental consanguinity status, the Human Phenotype Ontology (HPO) terms related to the reported clinical symptoms of patients, EDX findings, identified variants in genes with classification, and the MIM phenotype number, based on the Online Mendelian Inheritance in Man (OMIM) database. A summary of the genetic findings are presented in Table 1, including the involved genes, number of affected patients, number of novel variants, ACMG variant classifications, inheritance patterns, and associated OMIM phenotypes.

**Table 1:** Summary of genetic findings. Summary of the 107 WES probands across 54 genes. For each gene, the table shows the number of affected patients, the number of novel variants identified, ACMG variant classifications, mode of inheritance, and the associated OMIM phenotype(s)

Gene	Patients	Novel variants	Pathogenic/Likely pathogenic (*n*)	VUS (*n*)	Inheritance	Associated OMIM phenotype
*ACOX1*	1	1		1	AD	Mitchell syndrome (618960)
*ADPRS*	2	1	1 LP / 1 P	1	AR	Neurodegeneration, childhood-onset, stress-induced, with variable ataxia and seizures (618170)
*ARSA*	3	2	2 P / 2 LP		AR	Metachromatic Leukodystrophy (250100)
*C19ORF12*	2	1	1 LP	1	AR	Neurodegeneration with brain iron accumulation 4 (614298)
*CLP1*	3	2	1 P	2	AR	Pontocerebellar hypoplasia, type 10 (615803)
*CNTNAP1*	1	1	1 LP		AR	Hypomyelinating neuropathy, congenital, 3 (618186)
*CYP2U1*	2	1	1 P / 1 LP		AR	Spastic paraplegia 56, autosomal recessive (615030)
*DCTN1*	1	1		1	AD	Neuronopathy, distal hereditary motor, autosomal dominant 14 (607641)
*DHTKD1*	1	1		1	AD	Charcot-Marie-Tooth disease, axonal, type 2Q (615025)
*DNAJB2*	1	1	1 P		AR	Neuronopathy, distal hereditary motor, autosomal recessive 5 (614881)
*DYNC1H1*	2	2	1 P	1	AD	Cortical dysplasia, complex, with other brain malformations 13 (614563)
*ELOVL5*	1	1		1	AD	Spinocerebellar ataxia 38 (615957)
*FAM126A*	1	1	1 P		AR	Leukodystrophy, hypomyelinating, 5 (610532)
*FDXR*	3	2	1 LP	2	AR	Multiple mitochondrial dysfunctions syndrome 9B (620887)
*FLVCR1*	2	1	1 LP	1	AR	Retinopathy-sensory neuropathy syndrome (609033)
*GAN*	1	1		1	AR	Giant axonal neuropathy-1 (256850)
*GBF1*	1	1		1	AD	Charcot-Marie-Tooth disease, axonal, type 2GG (606483)
*HADHA*	1	0	1 P		AR	Mitochondrial trifunctional protein deficiency 1 (609015)
*HSPB1*	1	0	1 P		AD	Neuronopathy, distal hereditary motor, autosomal dominant 3 (608634)
*IBA57*	1	1		1	AR	Spastic paraplegia 74, autosomal recessive (616451)
*IGHMBP2*	4	1	2 P / 1 LP	1	AR	Neuronopathy, distal hereditary motor, type VI (604320); Charcot-Marie-Tooth disease, axonal, type 2 S (616155)
*JAG1*	1	0	1 P		AD	Charcot-Marie-Tooth disease, axonal, type 2HH (619574)
*KIF1A*	1	0	1 P		AD	NESCAV syndrome (614255)
*KIF1B*	2	2		2	AD	Charcot-Marie-Tooth disease, type 2A1 (118210)
*KIF5A*	1	1		1	AD	Spastic paraplegia 10, autosomal dominant (604187)
*MCM3AP*	1	1		1	AR	Peripheral neuropathy, autosomal recessive, with or without impaired intellectual development (618124)
*MFF*	2	2	2 P		AR	Encephalopathy due to defective mitochondrial and peroxisomal fission 2 (617086)
*MFN2*	2	1	2 P		AD	Charcot-Marie-Tooth disease, axonal, type 2A2A (609260)
*MFN2*	3	2	1 LP / 1 P	2	AR	Charcot-Marie-Tooth disease, axonal, type 2A2B (617087)
*MME*	2	1	1 P	1	AD	Charcot-Marie-Tooth disease, axonal, type 2T (617017); Spinocerebellar ataxia 43 (617018)
*MME*	3	2	3 P		AR	Charcot-Marie-Tooth disease, axonal, type 2T (617017)
*MORC2*	2	2		2	AD	Charcot-Marie-Tooth disease, axonal, type 2Z (616688); Developmental delay, impaired growth, dysmorphic facies, and axonal neuropathy (619090)
*MPV17*	1	0	1 LP	1	AR	Charcot-Marie-Tooth disease, axonal, type 2EE (618400)
*MPZ*	1	1		1	AD	Charcot-Marie-Tooth disease, type 1B (118200)
*MTMR2*	2	2	2 P		AR	Charcot-Marie-Tooth disease, type 4B1 (601382)
*NEFH*	2	2	1 LP / 1 P		AD	Charcot-Marie-Tooth disease, axonal, type 2CC (616924)
*PDXK*	1	0	1 P		AR	Neuropathy, hereditary motor and sensory, type VIC, with optic atrophy (618511)
*PIEZO2*	2	2		2	AR	Arthrogryposis, distal, with impaired proprioception and touch (617146)
*POLG*	2	0	2 P		AR	Mitochondrial recessive ataxia syndrome (includes SANDO and SCAE) (607459); Mitochondrial DNA depletion syndrome 4B (MNGIE type) (613662)
*PRX*	3	1	2 P / 1 LP		AR	Charcot-Marie-Tooth disease, type 4 F (614895)
*RETREG1*	1	0	1LP		AR	Neuropathy, hereditary sensory and autonomic, type IIB (613115)
*RNF170*	1	0	1 P		AR	Spastic paraplegia 85, autosomal recessive (619686)
*SACS*	11	8	7 LP / 4 P		AR	Spastic ataxia, Charlevoix-Saguenay type (270550)
*SBF2*	1	1	1 LP		AR	Charcot-Marie-Tooth disease, type 4B2 (604563)
*SCN11A*	1	1		1	AD	Neuropathy, hereditary sensory and autonomic, type VII (615548)
*SCN9A*	1	1	1 LP		AR	Neuropathy, hereditary sensory and autonomic, type IID (243000)
*SETX*	1	0	1 P		AD	Amyotrophic lateral sclerosis 4, juvenile (602433)
*SETX*	2	2	1 LP	1	AR	Spinocerebellar ataxia, autosomal recessive, with axonal neuropathy 2 (606002)
*SH3TC2*	1	1		1	AD	Mononeuropathy of the median nerve, mild (613353)
*SH3TC2*	4	3	1P/ 2LP	1	AR	Charcot-Marie-Tooth disease, type 4 C (601596)
*SNAP29*	1	0	1 P		AR	Cerebral dysgenesis, neuropathy, ichthyosis, and palmoplantar keratoderma syndrome (609528)
*SPG11*	5	3	3 P	2	AR	Spastic paraplegia 11, autosomal recessive (604360); Charcot-Marie-Tooth disease, axonal, type 2 × (616668)
*SPTBN4*	3	3	1 P	2	AR	Neurodevelopmental disorder with hypotonia, neuropathy, and deafness (617519)
*SYNE1*	1	2		2	AR	Spinocerebellar ataxia, autosomal recessive 8 (610743)
*SYT2*	1	1		1	AD	Myasthenic syndrome, congenital, 7 A, presynaptic, and distal motor neuropathy (616040)
*TECPR2*	1	1	1 P		AR	Neuropathy, hereditary sensory and autonomic, type IX, with developmental delay (HSAN9) (615031)
*TYMP*	2	2	1 P / 1 LP		AR	Mitochondrial DNA depletion syndrome 1 (MNGIE type) (603041)
*VPS13D*	1	1		1	AR	Spinocerebellar ataxia, autosomal recessive 4 (607317)

*AR* autosomal recessive, *AD* autosomal dominant, *P* pathogenic, *LP* likely pathogenic, *VUS* variant of uncertain significance. The per-gene “Patients” column sums to 108 because one proband (P91) carries a pathogenic/likely pathogenic variant in two different genes (*SH3TC2* and *NEFH*) and is therefore counted once under each gene. For three patients (P6 in *ADPRS*; P48 in *MFN2*; P56 in *MPV17*), one allele of a compound heterozygous genotype in an allele met pathogenic/likely pathogenic criteria while the other allele was classified as VUS; these patients are therefore not counted among patients with a confirmed molecular diagnosis. For variants classified as VUS, the reported OMIM phenotype was included for reference to the known gene–disease association and was not considered a definitive molecular diagnosis

To better interpret the identified variants, we assessed each variant using population frequency data from Genome Aggregation Database (gnomAD), Exome Aggregation Consortium (ExAC), Iranome and Genome Asia (Online Resource). We also evaluated predicted pathogenicity using multiple in silico tools, including SIFT and MutationTaster. The corresponding results are summarized in Supplementary Table 2.

**Fig. 3:**
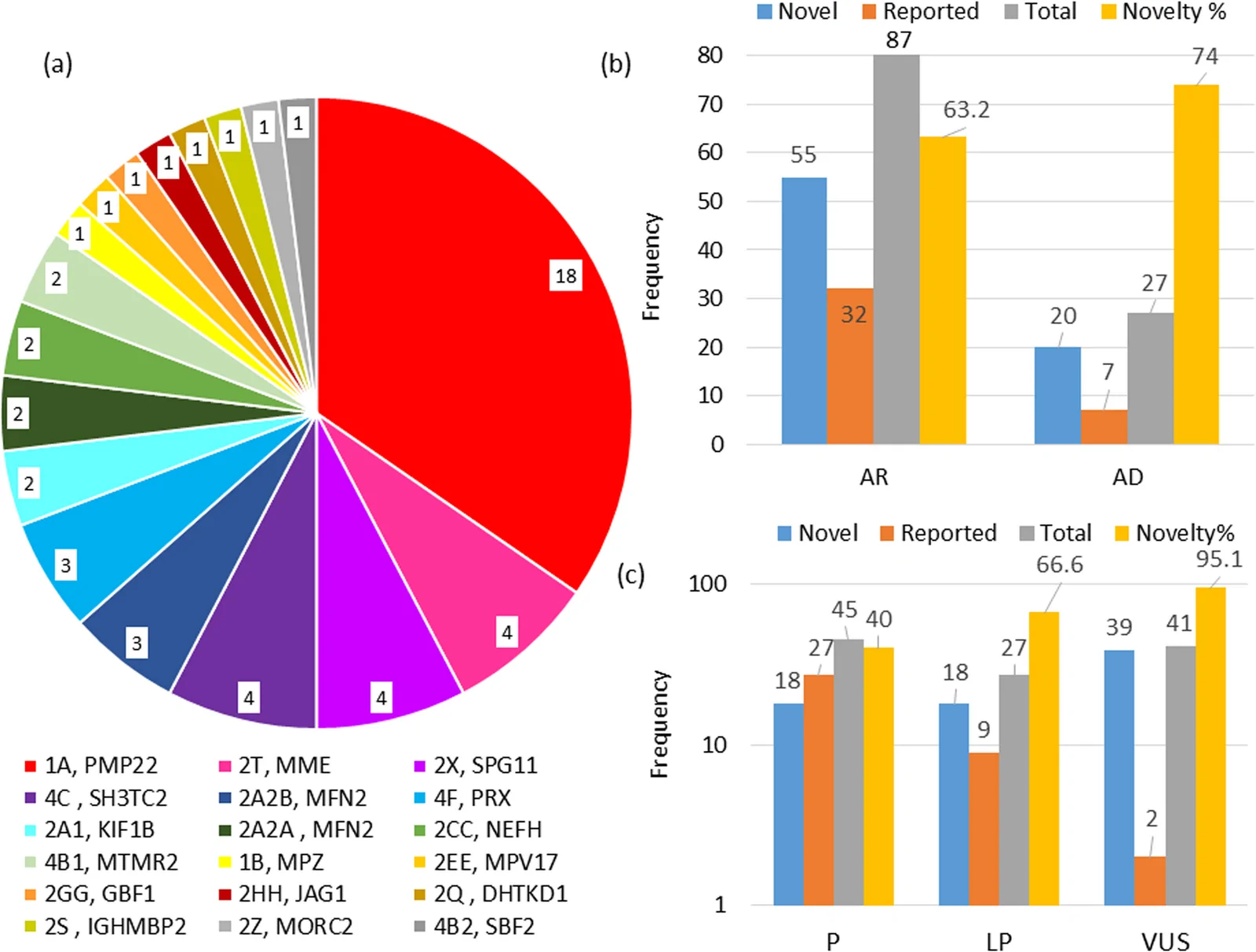
Summary of genetic findings in patients with IPN. (**a**) Distribution of Charcot-Marie-Tooth (CMT) subtypes identified by MLPA (*PMP22*, *n* = 18 CMT1A duplications) and WES (*n* = 34 identified genes across 33 patients; patient P91 carried two distinct CMT-associated results, CMT4C and CMT2CC). The single MLPA-diagnosed HNPP case (*PMP22* deletion) is not included, as HNPP represents a distinct clinical entity from CMT. (**b**) Inheritance patterns (autosomal recessive and autosomal dominant) and classification of variants as novel or previously reported. (**c**) Classification of identified variants based on pathogenicity

CMT disease was the most prevalent neuropathy subtype diagnosed in our cohort. We identified 18 different CMT subtypes in 41% of all patients (Fig. 3a). In total, we reported 107 patients with a candidate result in WES, with 113 different variants related to 54 genes. We reported 81 probands with AR inheritance (including 5 probands with compound heterozygous variants), 25 showed AD inheritance. Patients P35 and P36 presented with early IPN involvement in infancy, including respiratory distress and a weak cry, consistent with axonal sensorimotor neuropathy, although EDX reports for P36 were not available. After performing WES, 63.2% of AR-associated and 74.0% of AD-associated variants were novel (Fig. 3b). Patient 90 harbored a heterozygous *SH3TC2* variant (c.3839G>A; p.Trp1280*), classified as a VUS. Clinically, the patient presented with limping, difficulty climbing stairs, hand tremor, elevated serum CK levels, and electrophysiological evidence of a chronic generalized motor neuronopathy with axonal loss, a broader clinical symptom than the mild median mononeuropathy phenotype currently associated with *SH3TC2* in OMIM. Patient P91, a 27-year-old female from non-consanguineous parents, presented variants in two different CMT-associated genes with different inheritance patterns. She carried a novel homozygous likely pathogenic variant in *SH3TC2* (c.1691A>G; p.Asp564Gly) leading to CMT type 4C and a novel likely pathogenic heterozygous variant in *NEFH* c.646_653del (p.Leu216Glufs*61) leading to CMT type 2CC. Both CMT4C and CMT2CC typically present with distal muscle weakness.

Based on ACMG classification criteria [24], 63.7% (72/113) of the detected variants were classified as pathogenic or likely pathogenic, comprising 45 pathogenic and 27 likely pathogenic variants, and 50.0% (36/72) of these represented novel variants. This corresponds to an overall diagnostic yield of 62.6% (67/107) among the patients who underwent WES. Among the 107 patients who underwent WES, a molecular diagnosis was established in 31/47 (66.0%) patients with pure IPN phenotypes (CMT, HSN, dHMN) compared with 37/60 (61.7%) patients with complex neurogenetic phenotypes (HSP, SCA, spastic ataxia, mitochondrial disease, and neurodevelopmental/neurodegenerative disorders); this difference was not statistically significant (Fisher’s exact test, p = 0.69).” When MLPA-confirmed cases were included, the overall diagnostic yield for pure IPN phenotypes increased to 75.8% (50/66). Detailed ACMG evidence codes supporting the classification of all novel pathogenic and likely pathogenic variants are provided in Supplementary Table 3.

## Discussion

**Fig. 4:**
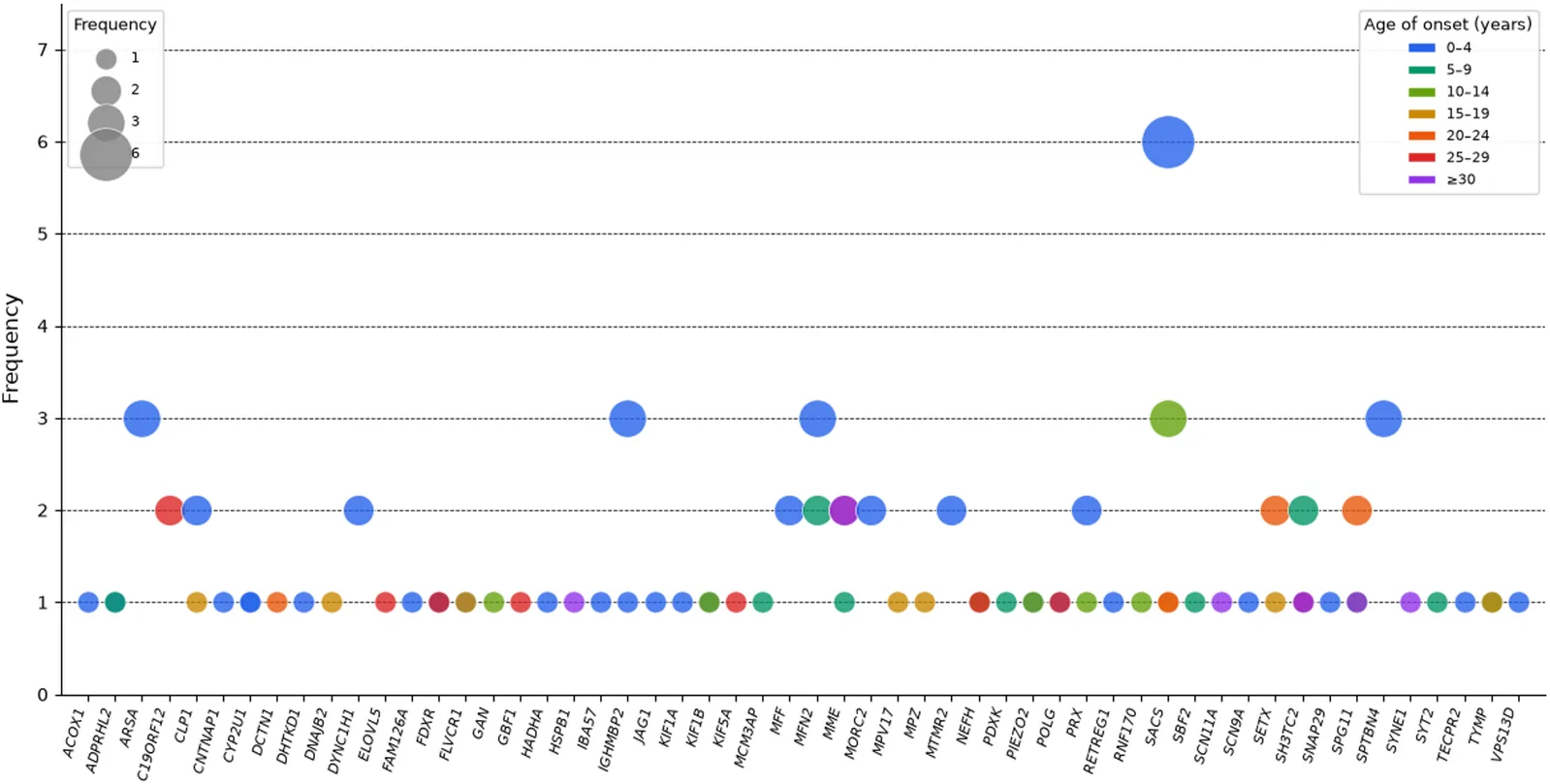
Distribution of disease associated genes identified by WES according to patient frequency and age of onset. Genes are plotted on the X-axis and the frequency of affected patients on the Y-axis. Bubble color indicates the age-of-onset group, and bubble size is proportional to the frequency of affected patients

Among patients referred for genetic testing, 19 were diagnosed with MLPA analysis targeting *PMP22* duplications and deletions, and 107 patients had a candidate variant in WES. These findings emphasize the utility of WES as a powerful diagnostic tool in the clinical evaluations. In this study, we reported 113 different variants and identified 36.2% (41/113) as VUS, with 95.1%, (39/41) of them also being novel (Fig. 3c). Although WES has become a valuable tool for genetic diagnostics, the frequent identification of VUS underscores a key limitation, as many variants require further functional evidence. The high proportion of novel VUS (95.1%) highlights the need for sequencing reports from underrepresented global populations. In addition, the high rate of novel variants emphasizes the need for functional studies [25]. Functional studies of VUS are needed for critical clinical applications, novel diagnoses, treatment and therapeutic approaches [26]. Another limitation of the study is that EDX data were unavailable for 10 of 107 WES-enrolled patients (9.3%); in these cases, phenotypic classification relied on clinical presentation and neurological examination alone. This lack of EDX confirmation may have led to phenotypic misclassification, particularly between CMT and dHMN, where the distinction relies on the relative degree of sensory versus motor involvement. In patients with complex neurogenetic phenotypes, the absence of EDX data may have similarly led to overestimation of the peripheral neuropathic component, as neurological symptoms in these cases can also arise from central nervous system involvement. Additionally, for compound heterozygous variants identified in the absence of parental samples, the trans configuration of the two variants could not be formally confirmed. These limitations should be considered when interpreting the phenotypic classifications reported in this cohort. Many patients in our cohort presented with IPN as a prominent or initial clinical feature. These cases were included as they reflect diagnostic challenges and highlight the utility of WES in identifying clinically overlapping phenotypes [5]. As shown in Fig. 4, the majority of patients exhibited early-onset forms of IPN, which was associated with a broad range of genetic etiologies. This finding underscores the predominance of early-onset neuropathies in the study population and supports the application of genetic testing in pediatric and early-onset adult cases. This diagnostic workflow should be considered when comparing the molecular subtype distribution of our cohort with studies applying uniform genomic testing, as it may have influenced the relative frequencies of specific CMT subtypes. Nevertheless, this approach reflects routine clinical practice and was intended to optimize diagnostic efficiency.

Among the genes identified in this cohort, *SACS* was the most frequently implicated gene, accounting for 11 unrelated patients with AR spastic ataxia of Charlevoix–Saguenay (ARSACS). Although ARSACS was described in the French-Canadian population because of founder variants [27], subsequent studies demonstrated that *SACS*-related disease occurs worldwide and exhibits allelic heterogeneity, particularly in populations with a high prevalence of consanguinity [28]. To date, no founder variants has been established in the Iranian population, and only a limited number of genetically confirmed Iranian ARSACS cases have been reported [29, 30]. Reports from neighboring Middle Eastern countries remain relatively scarce and have primarily described individual families harboring distinct *SACS* pathogenic variants, with no recurrent founder variant reported to date [31]. In our cohort, eight different pathogenic or likely pathogenic *SACS* variants were identified, seven of which were novel. Two unrelated patients (P78 and P80) carried the same novel variant, c.13288C>T (p.Gln4430*). Although this observation may raise the possibility of a founder variant, no conclusion can be drawn in the absence of haplotype analysis or identity-by-descent studies. Future studies involving haplotype-based analyses will be necessary to determine whether this recurrence reflects a shared ancestral origin or the enrichment of rare recessive alleles within this highly consanguineous population. Another finding in our cohort was patient P91, who carried pathogenic and likely pathogenic variants in *NEFH* and *SH3TC2*, respectively, which have different inheritance patterns. These two genes have been independently recognized as causes of IPNs through distinct biological mechanisms. Biallelic *SH3TC2* variants are associated with AR demyelinating CMT4C, whereas heterozygous *NEFH* variants cause AD axonal CMT2CC [32, 33]. The EDX findings in this patient demonstrated axonal sensorimotor neuropathy; however, in the presence of variants the EDX findings are insufficient to determine the relative contribution of each variant. Although a digenic contribution cannot be excluded, the currently available clinical and molecular evidence does not allow definitive confirmation of a combined pathogenic mechanism in this patient. A key strength of this study is the analysis of a population with a high rate of consanguineous marriages. Beyond the high prevalence of consanguinity, this cohort provides valuable insight into the genetic architecture of IPNs in Northeast Iran, a population that remains underrepresented in genomic studies. Although previous studies have investigated inherited neuropathies in Iranian patients, most have focused on specific disease subgroups, individual genes, or relatively small cohorts [34–36]. Compared with international populations, these cohorts are enriched for recessive neuropathies and provide opportunity to expand the genetic spectrum of IPNs. In contrast to European cohorts, where AD forms are more common, our cohort showed a higher proportion of AR neuropathies. This characteristic increases the detection of AR conditions and facilitates the identification of rare and novel biallelic variants. Advancements in NGS technologies have improved the diagnostic landscape of Mendelian disorders. Previous studies have similarly reported high diagnostic yields using WES in patients with IPNs [37, 38]. A study showed that the diagnosis of neuropathy from NGS testing, after exclusion of *PMP22* duplication, increases from 29% to 39.8% [39]. Following inclusion of the *PMP22* MLPA panel, it was reported to increase to 63.6% in a cohort study of 94 patients [40]. Ceyla et al. reported an 80% diagnostic yield from WES among 64 patients with hereditary neuropathy disease [41]. In the study by Senderek et al., a detection rate of 34.8% was reported from WES testing. This study was focused on axonal neuropathies of middle-aged patients [42]. Felice et al. reported a diagnostic yield of 10.7% following the use of WES on 108 patients with hereditary neuropathy [43]. In a limited number of studies, the diagnostic rate of whole genome sequencing has been reported. For example, a diagnostic rate of 31.8% in 233 patients was reported in the 100,000 genomes project [44]. While whole genome sequencing and long-read sequencing technologies have demonstrated improved diagnostic performance in specific contexts, particularly through the detection of structural variants, repeat expansions, and non-coding pathogenic variants [44], these approaches were not routinely available within the diagnostic workflow of the present cohort. Nevertheless, WES remains a cost-effective and widely accessible genomic testing strategy in many clinical settings, particularly in resource-limited regions [45]. The heterogeneity and clinical overlap are often observed in the major IPN related diseases, including CMT, dHMN, HSAN, HSP, and various types of metabolic neuropathies [46, 47]. As new gene-disease associations are discovered, reanalysis of raw sequencing data can uncover previously unrecognized pathogenic variants. This flexibility is essential for advancing both research and personalized medicine [48]. While targeted NGS panels for IPN continue to expand, they are unlikely to fully capture the growing list of IPN-associated genes. A multi-step diagnostic strategy is, therefore, recommended. For example, in cases of demyelinating neuropathy, testing for *PMP22* duplications, responsible for approximately 70% of such cases, should be performed before more extensive evaluations such as WES [48]. The estimated prevalence of IPNs in the general population is approximately 0.016% (1 in 6000) [49], but studies from Middle Eastern and African cohorts have reported different estimates [8]. While neuropathy-related risk factors have increased over recent decades, it remains unclear whether this has directly contributed to a rise in late-onset incidence [8]. A major challenge in epidemiological studies is the absence of standardized diagnostic criteria. Currently, the diagnosis is based on a combination of clinical symptoms and abnormal findings from nerve conduction studies [50]. Integrating detailed phenotyping with genetic test results represents a critical path in both diagnosis and population-level research [51].

## Conclusion

This study underscores the value of using WES in the genetic diagnosis of heterogeneous diseases such as IPNs in northeastern Iran. The identification of both known and novel variants broadens the molecular spectrum of IPNs and improves the genetic diagnosis of affected families. These findings provide insights into the genetic landscape of IPNs in an underrepresented population. They also support the integration of WES into the routine evaluation of patients with suspected IPNs and provide a foundation for future studies aimed at improving variant interpretation, genetic counseling, and precision medicine strategies.

## Supplementary Information

Below is the link to the electronic supplementary material.

Supplementary Material 1 (DOCX 110 KB)

Supplementary Material 2 (DOCX 39.3 KB)

Supplementary Material 3 (DOCX 20.8 KB)

## Data Availability

Variants have been submitted to ClinVar under the identifiers SUB15457538, SUB15457632, SUB15457695, SUB15457700, SUB15457701, SUB15457703, SUB15457707, SUB15457836, and SUB15457850.
