# Supplementary material for: Expanding the genetic spectrum of inherited peripheral neuropathies in complex neurogenetic disorders using whole-exome sequencing
Source: Neurogenetics. 2026 Oct 29;27(1):85. doi: 10.1007/s10048-026-00952-w (PMC13719752; doi:10.1007/s10048-026-00952-w)
Supplement: Supplementary file 1 [file 10048_2026_952_MOESM1_ESM.docx]

**Supplementary Table. 1** Summary of age at onset, parental consanguinity, EDX findings, and identified variants.

| **Patients** | **Gender** | **Consanguinity** | **Age onset** | **Phenotype and HPO** | **EDX Report** | **Gene (RefSeq ID)** | **Variant, Zygosity, Novel/Reported** | **Class** | **Associated OMIM phenotype** |
| --- | --- | --- | --- | --- | --- | --- | --- | --- | --- |
| **P1** | M^*^ | +^*^ | Infancy | Retinopathy (HP:0000488), Gait disturbance (HP:0001288) | Distal sensory polyneuropathy | *FLVCR1* (NM_014053.4) | c.1_18del (p.Met1?) HOM^*^, N^*^ | LP^*^ | Retinopathy-sensory neuropathy syndrome (609033), (AR^*^) |
| **P2** | F^*^ | + | 4y | Retinopathy (HP:0000488), Gait disturbance (HP:0001288) | Distal sensory polyneuropathy | *FLVCR1* (NM_014053.4) | c.502C>G (p.Leu168Val)  HOM, R^*^ [1] | VUS^*^ | Retinopathy-sensory neuropathy syndrome (609033), (AR) |
| **P3** | M | -^*^ | Infancy | Muscle weakness (HP:0001324) | Peripheral neuropathy | *KIF1B* (NM_001365953.1) | c.2756T>G (p.Leu919Arg) HET^*^, N | VUS | Charcot-Marie-Tooth disease, type 2A1 (118210), (AD^*^) |
| **P4** | M | - | 14y | Pes cavus (HP:0001761), Muscle weakness (HP:0001324), Hammertoe (HP:0001765) | Distal limb muscle weakness due to peripheral neuropathy | *KIF1B* (NM_001365953.1) | c.1981G>A (p.Ala661Thr) HET, N | VUS | Charcot-Marie-Tooth disease, type 2A1 (118210), (AD) |
| **P5** | M | - | Infancy | Seizure (HP:0001250), Peripheral neuropathy (HP:0009830), Inability to walk (HP:0002540), Absent speech (HP:0001344) | Peripheral neuropathy | *ACOX1* (NM_004035.7) | c.1674A>T (p.Leu558Phe) HET, N | VUS | Mitchell syndrome (618960), (AD) |
| **P6** | F | - | 7y | Muscle weakness (HP:0001324) | Sensorimotor axonal neuropathy | *ADPRS* (NM_017825.3) | c.530C>T (p.Ser177Leu)  HET, R [2] | LP | Neurodegeneration, childhood-onset, stress-induced, with variable ataxia and seizures (618170), (AR) |
|  |  |  |  |  |  |  | c.523C>T (p.Arg175Trp) HET, N | VUS |  |
| **P7** | F | + | 2y | Gait ataxia (HP:0002066) | Normal | *ADPRS* (NM_017825.3) | c.235A>C (p.Thr79Pro)  HOM, R [2] | P^*^ | Neurodegeneration, childhood-onset, stress-induced, with variable ataxia and seizures (618170), (AR) |
| **P8** | F | + | Infancy | Leukodystrophy (HP:0002415); Peripheral neuropathy (HP:0009830) | Chronic sensorimotor polyneuropathy with demyelinating features | *ARSA* (NM_000487.6 ) | c.938G>C (p.Arg313Pro)  HOM, R [3] | LP | Metachromatic Leukodystrophy (250100), (AR) |
| **P9** | F | + | Infancy | Abnormality of movement (HP:0100022) | Demyelinating hereditary motor sensory neuropathy | *ARSA* (NM_000487.6 ) | c.1283C>G (p.Pro428Arg) HOM, N | LP | Metachromatic Leukodystrophy (250100), (AR) |
| **P10** | M | - | Infancy | Inability to walk (HP:0002540); Leukodystrophy (HP:0002415) | Demyelinating hereditary motor sensory neuropathy | *ARSA* (NM_000487.6) | c.979+1G>A (p.?)  HET, R [4] | P | Metachromatic Leukodystrophy (250100), (AR) |
|  |  |  |  |  |  |  | c.393del (p.Pro132Leufs*16)  HET, N | P |  |
| **P11** | F | + | 24y | Abnormality of movement (HP:0100022); Speech impairment (HP:0002167) | Sensory axonal neuropathy | *C19ORF12* (NM_031448.6) | c.-2C>T (p.?) HOM, R [5] | LP | Neurodegeneration with brain iron accumulation 4 (614298), (AR) |
| **P12** | M | + | 28y | Abnormality of movement (HP:0100022); Speech impairment (HP:0002167); Tremor (HP:0001337); Cerebral atrophy (HP:0002059) | Sensory axonal neuropathy | *C19ORF12* (NM_031448.6) | c.17A>G (p.Glu6Gly) HOM, N | VUS | Neurodegeneration with brain iron accumulation 4 (614298), (AR) |
| **P13** | M | + | Infancy | Seizure (HP:0001250); Inability to walk (HP:0002540) | N/A^*^ | *CLP1* (NM_006831.3) | c.419G>A (p.Arg140His)  HOM R [6, 7] | P | Pontocerebellar hypoplasia, type 10 (615803), (AR) |
| **P14** | M | + | Infancy | Seizure (HP:0001250); Inability to walk (HP:0002540); Microcephaly (HP:0000252) | N/A | *CLP1* (NM_006831.3) | c.410A>G (p.Tyr137Cys) HOM, N | VUS | Pontocerebellar hypoplasia, type 10 (615803), (AR) |
| **P15** | F | + | 18y | Cerebellar atrophy (HP:0001272); Muscle weakness (HP:0001324) | Axonal sensorimotor neuropathy | *CLP1* (NM_006831.3) | c.617G>A (p.Arg206His) HOM, N | VUS | Pontocerebellar hypoplasia, type 10 (615803), (AR) |
| **P16** | F | + | Utero | Seizure (HP:0001250); Abnormality of movement (HP:0100022); Respiratory distress (HP:0002098) | Hypomyelinating neuropathy | *CNTNAP1* (NM_003632.3) | c.3750C>A (p.Cys1250*) HOM, N | LP | Hypomyelinating neuropathy, congenital, 3 (618186), (AR) |
| **P17** | M | + | 3y | Spastic paraplegia (HP:0001258) | Axonal neuropathy | *CYP2U1* (NM_183075.3) | c.947A>T (p.Asp316Val)  HOM, R [8] | P | Spastic paraplegia 56, autosomal recessive (615030), (AR) |
| **P18** | F | + | Infancy | Gait disturbance (HP:0001288) | Axonal neuropathy | *CYP2U1* (NM_183075.3) | c.491-2A>G (p.?) HOM, N | LP | Spastic paraplegia 56, autosomal recessive (615030), (AR) |
| **P19** | M | + | 25y | Distal muscle weakness (HP:0002460), Elevated circulating creatine kinase concentration (HP:0003236) | Distal motor neuropathy | *DCTN1* (NM_004082.5) | c.2090G>A (p.Arg697His) HET, N | VUS | Neuronopathy, distal hereditary motor, autosomal dominant 14 (607641), (AD) |
| **P20** | F | - | 5y | Muscle weakness (HP:0001324), Elevated circulating creatine kinase concentration (HP:0003236), Abnormal finger morphology (HP:0010049) | Chronic distal sensorimotor polyneuropathy | *DHTKD1* (NM_018706.7) | c.2110G>T (p.Ala704Ser) HET, N | VUS | Charcot-Marie-Tooth disease, axonal, type 2Q (615025), (AD) |
| **P21** | M | + | 16y | Gait disturbance (HP:0001288) | Axonal sensorimotor neuropathy | *DNAJB2* (NM_006736.6) | c.159T>G (p.Tyr53*) HOM, N | P | Neuronopathy, distal hereditary motor, autosomal recessive 5 (614881), (AR) |
| **P22** | M | + | Infancy | Seizure (HP:0001250); Delayed ability to walk (HP:0031936); Absent speech (HP:0001344); developmental delay (HP:0001263); Toe walking (HP:0040083) | N/A | *DYNC1H1* (NM_001376.5) | c.10007A>T (p.Lys3336Met) HET, N | P | Cortical dysplasia, complex, with other brain malformations 13 (614563), (AD) |
| **P23** | M | + | Infancy | Cerebral atrophy (HP:0002059); Microcephaly (HP:0000252); Developmental delay (HP:0001263) | N/A | *DYNC1H1* (NM_001376.5) | c.5288A>C (p.Glu1763Ala) HET, N | VUS | Cortical dysplasia, complex, with other brain malformations 13 (614563), (AD) |
| **P24** | F | - | 28y | Gait disturbance (HP:0001288) | Axonal neuropathy | *ELOVL5* (NM_021814.5) | c.871G>T (p.Val291Leu) HET, N | VUS | Spinocerebellar ataxia 38 (615957), (AD) |
| **P25** | F | + | Infancy | Inability to walk (HP:0002540); Developmental delay (HP:0001263); Cataract (HP:0000518) | Peripheral neuropathy | *FAM126A* (NM_032581.4) | c.229C>T (p.Gln77*) HOM, N | P | Leukodystrophy, hypomyelinating, 5 (610532), (AR) |
| **P26** | F | + | 5y | Seizure (HP:0001250); Abnormality of movement (HP:0100022); Visual impairment (HP:0000505); Hearing impairment (HP:0000365) | Sensorimotor peripheral neuropathy | *FDXR* (NM_024417.5) | c.463C>T (p.Arg155Trp)  HOM, R [9] | LP | Multiple mitochondrial dysfunctions syndrome 9B (620887), (AR) |
| **P27** | M | + | Infancy | Seizure (HP:0001250); Abnormality of movement (HP:0100022); Microcephaly (HP:0000252); Developmental delay (HP:0001263); Joint contracture (HP:0034392) | Sensorimotor peripheral neuropathy | *FDXR* (NM_024417.5) | c.773A>G (p.Asp258Gly) HOM, N | VUS | Multiple mitochondrial dysfunctions syndrome 9B (620887), (AR) |
| **P28** | M | - | 28y | Abnormality of movement (HP:0100022); Visual impairment (HP:0000505); Hearing impairment (HP:0000365); Muscle weakness (HP:0001324) | Sensorimotor peripheral neuropathy | *FDXR* (NM_024417.5) | c.247G>A (p.Ala83Thr) HOM, N | VUS | Multiple mitochondrial dysfunctions syndrome 9B (620887), (AR) |
| **P29** | F | - | 13y | Abnormality of movement (HP:0100022) | Sensory and motor axonal neuropathy | *GAN* (NM_022041.4) | c.616G>A (p.Asp206Asn) HOM, N | VUS | Giant axonal neuropathy-1 (256850), (AR) |
| **P30** | F | + | 27y | Abnormality of movement (HP:0100022) | Chronic sensorimotor distal polyneuropathy with axonal features | *GBF1* (NM_001377137.1) | c.2439T>G (p.Cys813Trp) HET, N | VUS | Charcot-Marie-Tooth disease, axonal, type 2GG (606483), (AD) |
| **P31** | M | + | 1y | Delayed ability to walk (HP:0031936); Gait disturbance (HP:0001288); Limitation of neck motion (HP:0005986) | Sensorimotor axonopathy | *HADHA* (NM_000182.5) | c.2026C>T (p.Arg676Cys)  HOM, R [10] | P | Mitochondrial trifunctional protein deficiency 1 (609015), (AR) |
| **P32** | F | - | 55y | Weakness of facial musculature (HP:0030319); Muscle weakness (HP:0001324) | Chronic distal sensorimotor polyneuropathy | *HSPB1* (NM_001540.5) | c.407G>T (p.Arg136Leu)  HET, R [11] | P | Neuronopathy, distal hereditary motor, autosomal dominant 3 (608634), (AD) |
| **P33** | F | + | Infancy | Delayed ability to walk (HP:0031936); Delayed ability to sit (HP:0025336) | N/A | *IBA57* (NM_001010867.4 ) | c.1033G>A (p.Ala345Thr) HOM, N | VUS | Spastic paraplegia 74, autosomal recessive (616451), (AR) |
| **P34** | F | + | Infancy | Delayed ability to walk (HP:0031936); Delayed ability to sit (HP:0025336); Abnormality of movement (HP:0100022); Respiratory distress (HP:0002098) | Axonal sensorimotor neuropathy | *IGHMBP2* (NM_002180.3) | c.1737C>A (p.Phe579Leu)  HOM, R [12] | LP | Neuronopathy, distal hereditary motor, type VI (604320), (AR) |
| **P35** | F | + | Infancy | Abnormality of movement (HP:0100022) | Axonal sensorimotor neuropathy | *IGHMBP2* (NM_002180.3) | c.2362C>T (p.Arg788*)  HOM, R [13] | P | Charcot-Marie- Tooth disease, axonal, type 2S (616155), (AR) |
| **P36** | F | + | Infancy | Abnormality of movement (HP:0100022) | N/A | *IGHMBP2* (NM_002180.3) | c.1708C>T (p.Arg570*)  HOM, R [14] | P | Neuronopathy, distal hereditary motor, type VI (604320), (AR) |
| **P37** | F | + | 1y | Abnormality of movement (HP:0100022); Abnormality of the skeletal system (HP:0000924); Muscle weakness (HP:0001324) | Axonal sensorimotor neuropathy | *IGHMBP2* (NM_002180.3) | c.509T>C (p.Leu170Pro) HOM, N | VUS | Neuronopathy, distal hereditary motor, type VI (604320), (AR) |
| **P38** | F | + | Infancy | Delayed ability to sit (HP:0025336); Hypotonia (HP:0001252); Abnormality of movement (HP:0100022); Ptosis (HP:0000508) | Axonal peripheral sensorimotor neuropathy | *JAG1* (NM_000214.3) | c.1666G>T (p.Glu556*) HET, R [15] | P | Charcot-Marie-Tooth disease, axonal, type 2HH (619574), (AD) |
| **P39** | M | + | 5y | Seizure (HP:0001250); Inability to walk (HP:0002540); Abnormality of movement (HP:0100022); Absent speech (HP:0001344); Optic atrophy (HP:0000648) | Axonal peripheral neuropathy | *KIF1A* (NM_001244008.2) | c.643A>C (p.Ser215Arg)  HET, R [16] | P | NESCAV syndrome (614255), (AD) |
| **P40** | F | + | 30y | Abnormality of movement (HP:0100022); Muscle weakness (HP:0001324) | Axonal peripheral sensorimotor neuropathy | *KIF5A* (NM_004984.4) | c.1111C>T (p.Arg371Cys) HET, N | VUS | Spastic paraplegia 10, autosomal dominant (604187), (AD) |
| **P41** | M | + | 10y | Abnormality of movement (HP:0100022) | Chronic sensorimotor distal polyneuropathy with axonal features | *MCM3AP* (NM_003906.5) | c.2243T>A (p.Ile748Asn) HOM, N | VUS | Peripheral neuropathy, autosomal recessive, with or without impaired intellectual development (618124), (AR) |
| **P42** | F | + | Infancy | Spasticity (HP:0001257); Developmental delay (HP:0001263); Visual impairment (HP:0000505) | Peripheral neuropathy | *MFF* (NM_001277062.2) | c.159del (p.Pro54Argfs*6)  HOM, N | P | Encephalopathy due to defective mitochondrial and peroxisomal fission 2 (617086), (AR) |
| **P43** | M | + | Infancy | Inability to walk (HP:0002540); Abnormality of movement (HP:0100022); Developmental delay (HP:0001263); Hypotonia (HP:0001252) | Peripheral neuropathy | *MFF* (NM_001277062.2) | c.352-2A>C (p.?) HOM, N | P | Encephalopathy due to defective mitochondrial and peroxisomal fission 2 (617086), (AR) |
| **P44** | F | + | Utero | Muscle weakness (HP:0001324) | N/A | *MFN2* (NM_014874.4) | c.610G>C (p.Asp204His) HET, N | P | Charcot-Marie-Tooth disease, axonal, type 2A2A (609260), (AD) |
| **P45** | F | - | 5y | Distal muscle weakness (HP:0002460); Hypoesthesia (HP:0033988); Insomnia (HP:0100785); Muscle weakness (HP:0001324) | Peripheral neuropathy | *MFN2* (NM_014874.4) | c.1091G>T (p.Arg364Leu)  HET, R [17] | P | Charcot-Marie-Tooth disease, axonal, type 2A2A (609260), (AD) |
| **P46** | F | + | Infancy | Muscle weakness (HP:0001324) | Axonal neuropathy | *MFN2* (NM_014874.4) | c.1988G>A (p.Arg663His) HOM, N | VUS | Charcot-Marie-Tooth disease, axonal, type 2A2B (617087), (AR) |
| **P47** | M | + | Infancy | Muscle weakness (HP:0001324) | Axonal neuropathy | *MFN2* (NM_014874.4) | c.154G>A (p.Glu52Lys)  HOM R [18] | LP | Charcot-Marie-Tooth disease, axonal, type 2A2B (617087), (AR) |
| **P48** | M | - | 6y | Gait disturbance (HP:0001288); Muscle weakness (HP:0001324) | Axonal neuropathy | *MFN2* (NM_014874.4) | c.839G>A (p.Arg280His)  HET, R [19] | P | Charcot-Marie-Tooth disease, axonal, type 2A2B (617087), (AR) |
|  |  |  |  |  |  |  | c.1556G>A (p.Arg519His) HET, N | VUS |  |
| **P49** | M | + | 44y | Gait disturbance (HP:0001288) | Peripheral neuropathy | *MME* (NM_007289.4) | c.2081G>A (p.Trp694*) HOM, N | P | Charcot-Marie- Tooth disease, axonal, type 2T(617017), (AR) |
| **P50** | M | - | 26y | Gait disturbance (HP:0001288) | Peripheral neuropathy | *MME* (NM_007289.4) | c.1564C>T (p.Gln522*)  HOM, R [20] | P | Charcot-Marie- Tooth disease, axonal, type 2T (617017), (AR) |
| **P51** | F | + | 25y | Gait disturbance (HP:0001288); Muscle weakness (HP:0001324) | Peripheral neuropathy | *MME* (NM_007289.4) | c.838G>C (p.Glu280Gln) HET N | VUS | Charcot-Marie- Tooth disease, axonal, type 2T (617017), (AD) |
| **P52** | M | + | 7y | Gait disturbance (HP:0001288); Ataxia (HP:0001251) | Peripheral neuropathy | *MME* (NM_007289.4) | c.1564C>T (p.Gln522*) HET, R [20] | P | Spinocerebellar ataxia 43 (617018), (AD) |
| **P53** | F | - | 42y | Gait disturbance (HP:0001288); Elevated circulating creatine kinase concentration (HP:0003236) | Peripheral neuropathy | *MME* (NM_007289.4) | c.655-1G>C (p.?) HOM, N | P | Charcot-Marie- Tooth disease, axonal, type 2T (617017), (AR) |
| **P54** | M | + | Infancy | Inability to walk (HP:0002540); Hypotonia (HP:0001252); Muscle weakness (HP:0001324) | Peripheral neuropathy | *MORC2* (NM_001303256.3) | c.278A>C (p.Glu93Ala) HET, N | VUS | Charcot-Marie-Tooth disease, axonal, type 2Z (616688), (AD) |
| **P55** | M | + | Infancy | Developmental delay (HP:0001263); Toe walking (HP:0040083); Strabismus (HP:0000486); Urinary retention (HP:0000016); Feeding difficulties (HP:0011968) | Peripheral neuropathy | *MORC2* (NM_001303256.3) | c.610C>T (p.Leu204Phe) HET, N | VUS | Developmental delay, impaired growth, dysmorphic facies, and axonal neuropathy (619090), (AD) |
| **P56** | M | + | 19y | Muscle weakness (HP:0001324) | Chronic sensorimotor distal polyneuropathy with axonal features | *MPV17* (NM_002437.5) | c.263A>T (p.Lys88Met)  HET, R [21] | LP | Charcot-Marie-Tooth disease, axonal, type 2EE (618400), (AR) |
|  |  |  |  |  |  |  | c.265A>T (p.Met89Leu)  HET, R [21] | VUS |  |
| **P57** | M | - | 20y | Abnormality of movement (HP:0100022) | Peripheral neuropathy | *MPZ* (NM_001315491.2) | c.775G>T (p.Gly259Trp) HET, N | VUS | Charcot-Marie-Tooth disease, type 1B (118200), (AD) |
| **P58** | F | + | Infancy | Abnormality of movement (HP:0100022); Speech impairment (HP:0002167); Gait disturbance (HP:0001288); Dysphagia (HP:0002015) | Peripheral neuropathy | *MTMR2* (NM_016156.6) | c.638del (p.Leu213*) HOM, N | P | Charcot-Marie- Tooth disease, type 4B1 (601382), (AR) |
| **P59** | F | + | Infancy | Delayed ability to sit (HP:0025336); Abnormality of movement (HP:0100022); Gait disturbance (HP:0001288); Developmental delay (HP:0001263) | Chronic sensorimotor polyneuropathy with demyelinating features | *MTMR2* (NM_016156.6) | c.1164G>A (p.Trp388*) HOM, N | P | Charcot-Marie- Tooth disease, type 4B1 (601382), (AR) |
| **P60** | M | - | 15y | Gait disturbance (HP:0001288); Abnormality of the skeletal system (HP:0000924); Scoliosis (HP:0002650); Muscle weakness (HP:0001324) | Chronic sensorimotor distal polyneuropathy with axonal features | *NEFH* (NM_021076.4) | c.884-1G>T (p.?) HET, N | LP | Charcot-Marie-Tooth disease, axonal, type 2CC (616924), (AD) |
| **P61** | M | + | 12y | Gait disturbance (HP:0001288) | normal | *PIEZO2* (NM_001378183.1) | c.3544C>T (p.Arg1182Cys)  HOM, N | VUS | Arthrogryposis, distal, with impaired proprioception and touch (617146), (AR) |
| **P62** | F | + | Infancy | Inability to walk (HP:0002540); Abnormality of movement (HP:0100022); Muscle weakness (HP:0001324) | Sensory axonal neuropathy | *PIEZO2* (NM_001378183.1) | c.104T>C (p.Leu35Pro) HOM, N | VUS | Arthrogryposis, distal, with impaired proprioception and touch (617146), (AR) |
| **P63** | F | + | 4y | Gait disturbance (HP:0001288); Ptosis (HP:0000508) | Sensory ataxic neuropathy | *POLG* (NM_002693.3) | c.911T>G (p.Leu304Arg)  HOM, R [22] | P | Mitochondrial DNA depletion syndrome 4B (MNGIE type) (613662), (AR) |
| **P64** | F | + | 30y | Abnormality of movement (HP:0100022); Speech impairment (HP:0002167); Vertigo (HP:0002321) | Sensory axonal neuropathy | *POLG* (NM_002693.3) | c.2243G>C (p.Trp748Ser)  HOM, R [22] | P | Mitochondrial recessive ataxia syndrome (includes SANDO and SCAE) (607459), (AR) |
| **P65** | F | + | Infancy | Delayed ability to walk (HP:0031936); Gait disturbance (HP:0001288); Intellectual disability (HP:0001249) | Peripheral neuropathy | *PRX* (NM_181882.3) | c.2035C>T (p.Arg679*) HOM, R [23] | LP | Charcot-Marie- Tooth disease, type 4F (614895), (AR) |
| **P66** | F | + | Infancy | Inability to walk (HP:0002540) | Peripheral neuropathy | *PRX* (NM_181882.3) | c.985del (p.Ala329Glnfs*27) HOM, N | P | Charcot-Marie- Tooth disease, type 4F (614895), (AR) |
| **P67** | F | - | 14y | Gait disturbance (HP:0001288) | Peripheral neuropathy | *PRX* (NM_181882.3) | c.1390C>T (p.Arg464*)  HOM, R [23] | P | Charcot-Marie- Tooth disease, type 4F (614895), (AR) |
| **P68** | M | + | 2y | Seizure (HP:0001250); Gait disturbance (HP:0001288) | Sensory axonal neuropathy | *RETREG1* (NM_001034850.3) | c.433C>T (p.Gln145*)  HOM, R [24] | P | Neuropathy, hereditary sensory and autonomic, type IIB (613115), (AR) |
| **P69** | F | + | 15y | Gait disturbance (HP:0001288); Muscle weakness (HP:0001324) | Sensory axonal neuropathy | *RNF170* (NM_030954.4) | c. 304T>C (p.Cys102Arg)  HOM, R [25] | LP | Spastic paraplegia 85, autosomal recessive (619686), (AR) |
| **P70** | M | + | 2y | Ataxia (HP:0001251) | Peripheral neuropathy | *SACS* (NM_014363.6) | c.8107C>T (p.Arg2703Cys)  HOM, R [26] | P | Spastic ataxia, Charlevoix-Saguenay type (270550), (AR) |
| **P71** | F | + | 14y | Distal muscle weakness (HP:0002460); Gait disturbance (HP:0001288) | Peripheral neuropathy | *SACS* (NM_014363.6) | c.408_409del (p.Gln137Ilefs*46)  HOM, N | P | Spastic ataxia, Charlevoix-Saguenay type (270550), (AR) |
| **P72** | F | + | 5y | Abnormality of movement (HP:0100022) | Peripheral neuropathy | *SACS* (NM_014363.6) | c.5347_5348insT (p.Arg1783Metfs*5)  HOM, N | LP | Spastic ataxia, Charlevoix-Saguenay type (270550), (AR) |
| **P73** | F | - | 27y | Gait disturbance (HP:0001288) | Peripheral neuropathy | *SACS* (NM_014363.6) | c.9508C>T (p.Arg3170*)  HOM, R [26] | P | Spastic ataxia, Charlevoix-Saguenay type (270550), (AR) |
| **P74** | M | + | 2y | Ataxia (HP:0001251) | Peripheral neuropathy | *SACS* (NM_014363.6) | c.8880dup (p.Ser2961Ilefs*6)  HOM, N | LP | Spastic ataxia, Charlevoix-Saguenay type (270550), (AR) |
| **P75** | M | + | 24y | Abnormality of movement (HP:0100022) | Peripheral neuropathy | *SACS* (NM_014363.6) | c.826C>T (p.Arg276Cys)  HOM, R [26] | P | Spastic ataxia, Charlevoix-Saguenay type (270550), (AR) |
| **P76** | F | + | 15y | Abnormality of movement (HP:0100022) | Peripheral neuropathy | *SACS* (NM_014363.6) | c.11953G>T (p.Glu3985*) HOM, N | LP | Spastic ataxia, Charlevoix-Saguenay type (270550), (AR) |
| **P77** | M | + | 10y | Delayed ability to walk (HP:0031936); Gait disturbance (HP:0001288); Intellectual disability (HP:0001249); Muscle weakness (HP:0001324) | Peripheral neuropathy | *SACS* (NM_014363.6) | c.5346del (p.Phe1782Leufs*32)  HOM, N | LP | Spastic ataxia, Charlevoix-Saguenay type (270550), (AR) |
| **P78** | M | + | 2y | Ataxia (HP:0001251) | Peripheral neuropathy | *SACS* (NM_014363.6) | c.13288C>T (p.Gln4430*) HOM, N | LP | Spastic ataxia, Charlevoix-Saguenay type (270550), (AR) |
| **P79** | M | + | 1y | Delayed ability to walk (HP:0031936); Abnormality of movement (HP:0100022); Muscle weakness (HP:0001324) | Peripheral neuropathy | *SACS* (NM_014363.6) | c.2976_2982del (p.Lys993Phefs*2) HOM, N | LP | Spastic ataxia, Charlevoix-Saguenay type (270550), (AR) |
| **P80** | F | + | 2y | Speech impairment (HP:0002167); Ataxia (HP:0001251), Gait disturbance (HP:0001288) | Peripheral neuropathy | *SACS* (NM_014363.6) | c.13288C>T (p.Gln4430*)  HOM, N | LP | Spastic ataxia, Charlevoix-Saguenay type (270550), (AR) |
| **P81** | M | + | 9y | Gait disturbance (HP:0001288) | Peripheral neuropathy | *SBF2* (NM_030962.4) | c.1395+1G>A (p.?) HOM, N | LP | Charcot-Marie-Tooth disease, type 4B2 (604563), (AR) |
| **P82** | M | - | 33y | Speech impairment (HP:0002167); Gait disturbance (HP:0001288); Muscle weakness (HP:0001324) | Chronic generalized motor neuropathy | *SCN11A* (NM_001349253.2) | c.4610_4611del (p.Phe1537Cysfs*31)  HET, N | VUS | Neuropathy, hereditary sensory and autonomic, type VII (615548), (AD) |
| **P83** | F | + | Infancy | Abnormality of the skeletal system (HP:0000924); Pain insensitivity (HP:0007021) | Sensory axonal neuropathy | *SCN9A* (NM_001365536.1) | c.4963A>G (p.Met1655Val)  HOM, N | LP | Neuropathy, hereditary sensory and autonomic, type IID (243000), (AR) |
| **P84** | M | + | 22y | Delayed ability to walk (HP:0031936); Ataxia (HP:0001251) | Chronic motor polyneuropathy with axonal features | *SETX* (NM_015046.7) | c.5243del (p.Phe1748Serfs*3)  HOM, N | LP | Spinocerebellar ataxia, autosomal recessive, with axonal neuropathy 2 (606002), (AR) |
| **P85** | M | + | 15y | Abnormality of movement (HP:0100022) | Sensorimotor distal polyneuropathy with axonal features | *SETX* (NM_015046.7) | c.6846G>C (p.Gln2282His) HOM, N | VUS | Spinocerebellar ataxia, autosomal recessive, with axonal neuropathy 2 (606002), (AR) |
| **P86** | F | + | 30y | Abnormality of movement (HP:0100022); Speech impairment (HP:0002167); Tremor (HP:0001337); Gait disturbance (HP:0001288); Muscle weakness (HP:0001324) | Sensorimotor distal polyneuropathy with axonal features | *SETX* (NM_015046.7) | c.5320C>T (p.Gln1774*)  HET, R [27] | P | Amyotrophic lateral sclerosis 4, juvenile (602433), (AD) |
| **P87** | M | + | 10Y | Abnormality of movement (HP:0100022); Gait disturbance (HP:0001288) | Chronic generalized motor neuropathy | *SH3TC2* (NM_024577.4) | c.856G>C (p.Glu286Gln) HOM, N | VUS | Charcot-Marie-Tooth disease, type 4C (601596), (AR) |
| **P88** | M | + | 7y | Abnormality of movement (HP:0100022); Gait disturbance (HP:0001288); Muscle weakness (HP:0001324) | Chronic sensorimotor polyneuropathy with demyelinating features | *SH3TC2* (NM_024577.4) | c.2860C>T (p.Arg954*)  HOM, R [27] | P | Charcot-Marie-Tooth disease, type 4C (601596), (AR) |
| **P89** | M | + | 22y | Abnormality of movement (HP:0100022); Muscle weakness (HP:0001324) | Chronic sensorimotor polyneuropathy with demyelinating features | *SH3TC2* (NM_024577.4) | c.1691A>G (p.Asp564Gly) HOM, N | LP | Charcot-Marie-Tooth disease, type 4C (601596), (AR) |
| **P90** | F | - | 46y | Tremor (HP:0001337); Gait disturbance (HP:0001288); Muscle weakness (HP:0001324) | Chronic generalized motor neuropathy | *SH3TC2* (NM_024577.4) | c.3839G>A (p.Trp1280*) HET, N | VUS | Mononeuropathy of the median nerve, mild (613353), (AD) |
| **P91** | F | - | 27y | Abnormality of movement (HP:0100022); Gait disturbance (HP:0001288) | Axonal sensorimotor neuropathy | *SH3TC2* (NM_024577.4) | c.1691A>G (p.Asp564Gly) HOM, N | LP | Charcot-Marie-Tooth disease, type 4C (601596), (AR) |
|  |  |  |  |  |  | *NEFH* (NM_021076.4) | c.646_653del (p.Leu216Glufs*61) HET, N | P | Charcot-Marie-Tooth disease, axonal, type 2CC (616924), (AD) |
| **P92** | F | + | Infancy | Gait disturbance (HP:0001288); Developmental delay (HP:0001263); Feeding difficulties (HP:0011968) | Peripheral neuropathy | *SNAP29* (NM_004782.4) | c.487dup (p.Ser163Lysfs*6)  HOM, R [28] | P | Cerebral dysgenesis, neuropathy,ichthyosis, and palmoplantar keratoderma syndrome (609528), (AR) |
| **P93** | M | + | 14y | Abnormality of movement (HP:0100022); Gait disturbance (HP:0001288) | Axonal neuropathy | *SPG11* (NM_025137.4) | c.3143_3144dup (p.Asp1049Glnfs*5) HOM, R [29] | P | Charcot-Marie-Tooth disease, axonal, type 2X (616668), (AR) |
| **P94** | F | + | Infancy | Abnormality of movement (HP:0100022); Gait disturbance (HP:0001288); | Axonal neuropathy | *SPG11* (NM_025137.4) | c.250T>C (p.Phe84Leu) HOM, N | VUS | Charcot-Marie-Tooth disease, axonal, type 2X (616668), (AR) |
| **P95** | F | + | 35y | Gait disturbance (HP:0001288); | Axonal neuropathy | *SPG11* (NM_025137.4) | c.3075dup (p.Glu1026Argfs*4) HOM, R [29] | P | Charcot-Marie-Tooth disease, axonal, type 2X (616668), (AR) |
| **P96** | M | - | 20y | Gait disturbance (HP:0001288); Muscle weakness (HP:0001324), Cognitive impairment (HP:0100543) | Distal peripheral motor and sensory neuropathy | *SPG11* (NM_025137.4) | c.4001G>A (p.Arg1334Lys)  HOM, N | VUS | Spastic paraplegia 11, autosomal recessive (604360), (AR) |
| **P97** | F | + | 22y | Abnormality of movement (HP:0100022) | Axonal neuropathy | *SPG11* (NM_025137.4) | c.5616G>A (p.Trp1872*) HOM, N | P | Charcot-Marie-Tooth disease, axonal, type 2X (616668), (AR) |
| **P98** | F | + | Infancy | Delayed ability to sit (HP:0025336); Motor delay (HP:0001270); Absent speech (HP:0001344); Muscle weakness (HP:0001324) | Axonal peripheral neuropathy | *SPTBN4* (NM_020971.3) | c.1795del (p.Leu599Cysfs*21) HOM, N | P | Neurodevelopmental disorder with hypotonia, neuropathy, and deafness (617519), (AR) |
| **P99** | M | + | Infancy | Abnormality of movement (HP:0100022); Hypotonia (HP:0001252) | N/A | *SPTBN4* (NM_020971.3) | c.1789G>A (p.Ala597Thr) HOM, N | VUS | Neurodevelopmental disorder with hypotonia, neuropathy, and deafness (617519), (AR) |
| **P100** | F | + | Infancy | Muscle weakness (HP:0001324), ; Hypotonia (HP:0001252) | Axonal and demyelinating peripheral neuropathy | *SPTBN4* (NM_020971.3) | c.625T>C (p.Trp209Arg) HOM, N | VUS | Neurodevelopmental disorder with hypotonia, neuropathy, and deafness (617519), (AR) |
| **P101** | M | - | 30y | Abnormality of movement (HP:0100022); Gait disturbance (HP:0001288); Muscle weakness (HP:0001324) | Axonal peripheral neuropathy | *SYNE1* (NM_182961.4) | c.18620C>T (p.Thr6207Met) HET, N | VUS | Spinocerebellar ataxia, autosomal recessive 8 (610743), (AR) |
|  |  |  |  |  |  |  | c.5267G>A (p.Arg1756Lys) HET, N | VUS |  |
| **P102** | F | - | 10y | Hypotonia (HP:0001252); Muscle weakness (HP:0001324) | N/A | *SYT2* (NM_177402.5) | c.2T>A (p.Met1?)  HET, N | VUS | Myasthenic syndrome, congenital, 7A, presynaptic, and distal motor neuropathy, autosomal dominant (616040), (AD) |
| **P103** | M | + | Infancy | Delayed ability to walk (HP:0031936); Abnormality of movement (HP:0100022); Absent speech (HP:0001344); Microcephaly (HP:0000252) | N/A | *TECPR2* (NM_014844.5) | c.911G>A (p.Trp304*) HOM, N | P | Neuropathy, hereditary sensory and autonomic, type IX, with developmental delay (HSAN9) (615031), (AR) |
| **P104** | M | + | 20y | Abnormality of the gastrointestinal tract (HP:0011024); Muscle weakness (HP:0001324) | Sensorimotor neuropathy with axonal features | *TYMP* (NM_001953.5) | c.31_32insAACCGGG (p.Ala11Glufs*9) HOM, N | P | Mitochondrial DNA depletion syndrome 1(MNGIE type) (603041), (AR) |
| **P105** | M | + | 9y | Abnormality of movement (HP:0100022); Abnormal foot morphology (HP:0001760) | Sensorimotor axonal neuropathy | *TYMP* (NM_001953.5) | c.518T>A (p.Met173Lys) HOM, N | LP | Mitochondrial DNA depletion syndrome 1(MNGIE type) (603041), (AR) |
| **P106** | M | + | Infancy | Seizure (HP:0001250); Inability to walk (HP:0002540); Absent speech (HP:0001344); Developmental delay (HP:0001263) | Sensorimotor axonal neuropathy | *VPS13D* (NM_015378.4) | c.2961T>G (p.Phe987Leu) HOM, N | VUS | Spinocerebellar ataxia, autosomal recessive 4 (607317), (AR) |
| **P107** | F | + | 9y | Abnormality of movement (HP:0100022) | Axonal peripheral sensorimotor neuropathy | *PDXK* (NM_003681.4) | c.225T>A (p.Asn75Lys) HOM, R [30] | P | Neuropathy, hereditary motor and sensory, type VIC, with optic atrophy (618511), (AR) |

**Phenotypic information reported in this table is based on available EDX assessments and clinical observations. Variant classification was performed according to ACMG guidelines. MIM phenotype numbers are based on the OMIM database.**

***Abbreviations**: P: patient, EDX: Electrodiagnosis, NM: NCBI Reference Sequence (RefSeq) mRNA accession number, OMIM: Online Mendelian Inheritance in Man, M: male, F: female, (+): parents had consanguinity, (-): parents not related, N/A: Not Applicable, N: Novel variant, R: Reported variant, P: pathogenic, LP: likely pathogenic, VUS: Variants of Uncertain Significance, AR: Autosomal Recessive, AD: Autosomal Dominant. A designation of “normal” refers to normal EDX findings and does not exclude the presence of additional clinical symptoms. EDX descriptions were extracted from the original clinical reports, and detailed information was not available for all patients. For variants classified as VUS, the reported OMIM phenotype was included for reference to the known gene–disease association and was not considered a definitive molecular diagnosis.

References:

1.	Calame, D.G., et al., *Biallelic variation in the choline and ethanolamine transporter FLVCR1 underlies a severe developmental disorder spectrum.* Genet Med, 2025. **27**(1): p. 101273.

2.	Ghosh, S.G., et al., *Biallelic Mutations in ADPRHL2, Encoding ADP-Ribosylhydrolase 3, Lead to a Degenerative Pediatric Stress-Induced Epileptic Ataxia Syndrome.* Am J Hum Genet, 2018. **103**(3): p. 431-439.

3.	Yari, A., et al., *Identification of a Novel ARSA Gene Mutation Through High-Throughput Molecular Diagnosis Method in Two Girls with Late Infantile Metachromatic Leukodystrophy.* Neuromolecular Med, 2023. **25**(4): p. 563-572.

4.	Eng, B., et al., *Identification of nine novel arylsulfatase a (ARSA) gene mutations in patients with metachromatic leukodystrophy (MLD).* Hum Mutat, 2003. **22**(5): p. 418-9.

5.	Dusek, P., et al., *Brain iron and metabolic abnormalities in C19orf12 mutation carriers: A 7.0 tesla MRI study in mitochondrial membrane protein-associated neurodegeneration.* Mov Disord, 2020. **35**(1): p. 142-150.

6.	Cavusoglu, D., et al., *Evaluation of the Patients with the Diagnosis of Pontocerebellar Hypoplasia: A Multicenter National Study.* Cerebellum, 2024. **23**(5): p. 1950-1965.

7.	Morisaki, I., et al., *Modeling a human CLP1 mutation in mouse identifies an accumulation of tyrosine pre-tRNA fragments causing pontocerebellar hypoplasia type 10.* Biochem Biophys Res Commun, 2021. **570**: p. 60-66.

8.	Durand, C.M., et al., *CYP2U1 activity is altered by missense mutations in hereditary spastic paraplegia 56.* Hum Mutat, 2018. **39**(1): p. 140-151.

9.	Slone, J., et al., *Biallelic mutations in FDXR cause neurodegeneration associated with inflammation.* J Hum Genet, 2018. **63**(12): p. 1211-1222.

10.	Boutron, A., et al., *Comprehensive cDNA study and quantitative analysis of mutant HADHA and HADHB transcripts in a French cohort of 52 patients with mitochondrial trifunctional protein deficiency.* Mol Genet Metab, 2011. **103**(4): p. 341-8.

11.	Abati, E., et al., *Charcot-Marie-Tooth disease type 2F associated with biallelic HSPB1 mutations.* Ann Clin Transl Neurol, 2021. **8**(5): p. 1158-1164.

12.	Xie, Y., et al., *Genotype and phenotype distribution of 435 patients with Charcot-Marie-Tooth disease from central south China.* Eur J Neurol, 2021. **28**(11): p. 3774-3783.

13.	Cottenie, E., et al., *Truncating and missense mutations in IGHMBP2 cause Charcot-Marie Tooth disease type 2.* Am J Hum Genet, 2014. **95**(5): p. 590-601.

14.	Schottmann, G., et al., *Recessive truncating IGHMBP2 mutations presenting as axonal sensorimotor neuropathy.* Neurology, 2015. **84**(5): p. 523-31.

15.	Colliton, R.P., et al., *Mutation analysis of Jagged1 (JAG1) in Alagille syndrome patients.* Hum Mutat, 2001. **17**(2): p. 151-2.

16.	Lee, J.R., et al., *De novo mutations in the motor domain of KIF1A cause cognitive impairment, spastic paraparesis, axonal neuropathy, and cerebellar atrophy.* Hum Mutat, 2015. **36**(1): p. 69-78.

17.	Feely, S.M., et al., *MFN2 mutations cause severe phenotypes in most patients with CMT2A.* Neurology, 2011. **76**(20): p. 1690-6.

18.	Ando, M., et al., *Clinical and genetic diversities of Charcot-Marie-Tooth disease with MFN2 mutations in a large case study.* J Peripher Nerv Syst, 2017. **22**(3): p. 191-199.

19.	Chen, C.X., et al., *Genetic spectrum and clinical profiles in a southeast Chinese cohort of Charcot-Marie-Tooth disease.* Clin Genet, 2019. **96**(5): p. 439-448.

20.	Jamiri, Z., et al., *A nonsense mutation in MME gene associates with autosomal recessive late-onset Charcot-Marie-Tooth disease.* Mol Genet Genomic Med, 2022. **10**(5): p. e1913.

21.	Garone, C., et al., *MPV17 Mutations Causing Adult-Onset Multisystemic Disorder With Multiple Mitochondrial DNA Deletions.* Arch Neurol, 2012. **69**(12): p. 1648-51.

22.	Saha, D., et al., *Genetic heterogeneity and respiratory chain enzyme analysis in pediatric Indian patients with mitochondrial disorder: Report of novel variants in POLG1 gene and their functional implication using molecular dynamic simulation.* Mitochondrion, 2024. **76**: p. 101870.

23.	Choi, Y.J., et al., *Novel Compound Heterozygous Nonsense PRX Mutations in a Korean Dejerine-Sottas Neuropathy Family.* J Clin Neurol, 2015. **11**(1): p. 92-6.

24.	Çakar, A., et al., *Phenotypic features of RETREG1-related hereditary sensory autonomic neuropathy.* J Peripher Nerv Syst, 2023. **28**(3): p. 351-358.

25.	Wagner, M., et al., *Bi-allelic variants in RNF170 are associated with hereditary spastic paraplegia.* Nat Commun, 2019. **10**(1): p. 4790.

26.	Pilliod, J., et al., *New practical definitions for the diagnosis of autosomal recessive spastic ataxia of Charlevoix-Saguenay.* Ann Neurol, 2015. **78**(6): p. 871-86.

27.	Lerat, J., et al., *Implication of the SH3TC2 gene in Charcot-Marie-Tooth disease associated with deafness and/or scoliosis: Illustration with four new pathogenic variants.* J Neurol Sci, 2019. **406**: p. 116376.

28.	Sprecher, E., et al., *A mutation in SNAP29, coding for a SNARE protein involved in intracellular trafficking, causes a novel neurocutaneous syndrome characterized by cerebral dysgenesis, neuropathy, ichthyosis, and palmoplantar keratoderma.* Am J Hum Genet, 2005. **77**(2): p. 242-51.

29.	Xing, F. and J. Du, *Expansion of the mutation and phenotypic spectrum of hereditary spastic paraplegia.* Neurol Sci, 2022. **43**(8): p. 4989-4996.

30.	Keller, N., et al., *Hereditary polyneuropathy with optic atrophy due to PDXK variant leading to impaired Vitamin B6 metabolism.* Neuromuscul Disord, 2020. **30**(7): p. 583-589.
