# Supplementary material for: Expanding the genetic spectrum of inherited peripheral neuropathies in complex neurogenetic disorders using whole-exome sequencing
Source: Neurogenetics. 2026 Oct 29;27(1):85. doi: 10.1007/s10048-026-00952-w (PMC13719752; doi:10.1007/s10048-026-00952-w)
Supplement: Supplementary file 2 [file 10048_2026_952_MOESM2_ESM.docx]

**Supplementary Table 2. Population allele frequencies and in silico pathogenicity predictions for identified variants.**

| **Patient/ Gene/ Variant** | **MUT Assessor** | **SIFT** | **gnomAD (%)** | **Iranome (%)** | **ExAC (%)** | **GenomeAsia (%)** |
| --- | --- | --- | --- | --- | --- | --- |
| **P1** *FLVCR1* c.1_18del (p.Met1?) | N/A | N/A | 0 | 0 | 0 | 0 |
| **P2** *FLVCR1* c.502C>G (p.Leu168Val) | Med (2.53) | Uncertain (0.002) | 0.0001 | 0 | 0 | 0 |
| **P3** *KIF1B* c.2756T>G (p.Leu919Arg) | N/A | Deleterious (Supporting) (0) | 0 | 0 | 0 | 0 |
| **P4** *KIF1B* c.1981G>A (p.Ala661Thr) | N/A | Uncertain (0.063) | 0.0027 | 0 | 0.0075 | 0 |
| **P5** *ACOX1* c.1674A>T (p.Leu558Phe) | Hi (3.79) | Uncertain (0.002) | 0 | 0 | 0 | 0 |
| **P6** *ADPRHL2* c.530C>T (p.Ser177Leu) | Med (3.46) | Uncertain (0.003) | 0.0007 | 0 | 0 | 0 |
| **P6** *ADPRHL2* c.523C>T (p.Arg175Trp) | Med (2.56) | Uncertain (0.001) | 0.0014 | 0 | 0.0017 | 0 |
| **P7** *ADPRHL2* c.235A>C (p.Thr79Pro) | Hi (3.94) | Uncertain (0.003) | 0.0001 | 0 | 0 | 0 |
| **P8** *ARSA* c.938G>C (p.Arg313Pro) | N/A | Deleterious (Supporting) (0) | 0 | 0 | 0 | 0 |
| **P9** *ARSA* c.1283C>G (p.Pro428Arg) | N/A | Deleterious (Supporting) (0) | 0 | 0 | 0 | 0 |
| **P10** *ARSA* c.979+1G>A (p.?) | N/A | N/A | 0.0004 | 0 | 0 | 0 |
| **P10** *ARSA* c.393del (p.Pro132Leufs*16) | N/A | N/A | 0 | 0 | 0 | 0 |
| **P11** *C19ORF12* c.-2C>T (p.?) | Neutral (-0.35) | Supporting (0.102) | 0.0014 | 0 | 0.0008 | 0 |
| **P12** *C19ORF12* c.17A>G (p.Glu6Gly) | Lo (1.15) | Supporting (0.08) | 0.0001 | 0 | 0 | 0 |
| **P13** *CLP1* c.419G>A (p.Arg140His) | Med (2.47) | Uncertain (0.001) | 0.0018 | 0 | 0.0008 | 0 |
| **P14** *CLP1* c.410A>G (p.Tyr137Cys) | Med (3.41) | Deleterious (Supporting) (0) | 0 | 0 | 0 | 0 |
| **P15** *CLP1* c.617G>A (p.Arg206His) | Med (2.38) | Uncertain (0.013) | 0.0005 | 0 | 0 | 0 |
| **P16** *CNTNAP1* c.3750C>A (p.Cys1250*) | N/A | N/A | 0.0001 | 0 | 0 | 0 |
| **P17** *CYP2U1* c.947A>T (p.Asp316Val) | Hi (4.53) | Deleterious (Supporting) (0) | 0.0005 | 0 | 0 | 0 |
| **P18** *CYP2U1* c.491-2A>G (p.?) | N/A | N/A | 0 | 0 | 0 | 0 |
| **P19** *DCTN1* c.2090G>A (p.Arg697His) | Med (2.34) | Uncertain (0.002) | 0.0014 | 0 | 0.0049 | 0 |
| **P20** *DHTKD1* c.2110G>T (p.Ala704Ser) | Med (1.98) | Uncertain (0.006) | 0.0001 | 0 | 0 | 0 |
| **P21** *DNAJB2* c.159T>G (p.Tyr53*) | N/A | N/A | 0 | 0 | 0 | 0 |
| **P22** *DYNC1H1* c.10007A>T (p.Lys3336Met) | Hi (3.5) | Uncertain (0.001) | 0 | 0 | 0 | 0 |
| **P23** *DYNC1H1* c.5288A>C (p.Glu1763Ala) | Hi (3.55) | Uncertain (0.003) | 0 | 0 | 0 | 0 |
| **P24** *ELOVL5* c.871G>T (p.Val291Leu) | N/A | N/A | 0.001 | 0 | 0 | 0 |
| **P25** *FAM126A* c.229C>T (p.Gln77*) | N/A | N/A | 0 | 0 | 0 | 0 |
| **P26** *FDXR* c.463C>T (p.Arg155Trp) | N/A | Deleterious (Supporting) (0) | 0.0012 | 0 | 0.0008 | 0 |
| **P27** *FDXR* c.773A>G (p.Asp258Gly) | N/A | Uncertain (0.004) | 0 | 0 | 0 | 0 |
| **P28** *FDXR* c.247G>A (p.Ala83Thr) | N/A | Deleterious (Supporting) (0) | 0 | 0 | 0 | 0 |
| **P29** *GAN* c.616G>A (p.Asp206Asn) | Med (2.09) | N/A | 0 | 0 | 0 | 0 |
| **P30** *GBF1* c.2439T>G (p.Cys813Trp) | Med (2.92) | Uncertain (0.01) | 0 | 0 | 0 | 0 |
| **P31** *HADHA* c.2026C>T (p.Arg676Cys) | Hi (5.06) | Deleterious (Supporting) (0) | 0.0004 | 0 | 0.0025 | 0 |
| **P32** *HSPB1* c.407G>T (p.Arg136Leu) | Hi (4.12) | Deleterious (Supporting) (0) | 0.0002 | 0 | 0 | 0 |
| **P33** *IBA57* c.1033G>A (p.Ala345Thr) | Med (2.57) | Supporting (0.113) | 0.0317 | 0 | 0.0247 | 0 |
| **P34** *IGHMBP2* c.1737C>A (p.Phe579Leu) | Neutral (-1.42) | Supporting (0.111) | 0.0001 | 0 | 0 | 0 |
| **P35** *IGHMBP2* c.2362C>T (p.Arg788*) | N/A | N/A | 0.0026 | 0 | 0.0035 | 0 |
| **P36** *IGHMBP2* c.1708C>T (p.Arg570*) | N/A | N/A | 0.0008 | 0 | 0 | 0 |
| **P37** *IGHMBP2* c.509T>C (p.Leu170Pro) | Med (3.11) | Uncertain (0.006) | 0 | 0 | 0 | 0 |
| **P38** *JAG1* c.1666G>T (p.Glu556*) | N/A | N/A | 0 | 0 | 0 | 0 |
| **P39** *KIF1A* c.643A>C (p.Ser215Arg) | Hi (4.14) | Deleterious (Supporting) (0) | 0 | 0 | 0 | 0 |
| **P40** *KIF5A* c.1111C>T (p.Arg371Cys) | Med (2.74) | Deleterious (Supporting) (0) | 0.001 | 0 | 0 | 0 |
| **P41** *MCM3AP* c.2243T>A (p.Ile748Asn) | Lo (0.91) | Uncertain (0.002) | 0 | 0 | 0 | 0 |
| **P42** *MFF* c.159del (p.Pro54Argfs*6) | N/A | N/A | 0 | 0 | 0 | 0 |
| **P43** *MFF* c.352-2A>C (p.?) | N/A | N/A | 0 | 0 | 0 | 0 |
| **P44** *MFN2* c.610G>C (p.Asp204His) | Med (2.8) | Uncertain (0.001) | 0 | 0 | 0 | 0 |
| **P45** *MFN2* c.1091G>T (p.Arg364Leu) | Med (3.04) | Uncertain (0.002) | 0 | 0 | 0 | 0 |
| **P46** *MFN2* c.1988G>A (p.Arg663His) | Med (1.94) | Uncertain (0.02) | 0.0031 | 0 | 0.0017 | 0 |
| **P47** *MFN2* c.154G>A (p.Glu52Lys) | Med (2.58) | Uncertain (0.002) | 0 | 0 | 0 | 0 |
| **P48** *MFN2* c.839G>A (p.Arg280His) | Med (3.24) | Deleterious (Supporting) (0) | 0.0002 | 0 | 0.0008 | 0 |
| **P48** *MFN2* c.1556G>A (p.Arg519His) | Lo (1.83) | Supporting (0.123) | 0.0003 | 0 | 0.0008 | 0 |
| **P49** *MME* c.2081G>A (p.Trp694*) | N/A | N/A | 0.0001 | 0 | 0 | 0 |
| **P50** *MME* c.1564C>T (p.Gln522*) | N/A | N/A | 0.0001 | 0 | 0 | 0 |
| **P51** *MME* c.838G>C (p.Glu280Gln) | Med (3) | Deleterious (Supporting) (0) | 0 | 0 | 0 | 0 |
| **P52** *MME* c.1564C>T (p.Gln522*) | N/A | N/A | 0.0001 | 0 | 0 | 0 |
| **P53** *MME* c.655-1G>C (p.?) | N/A | N/A | 0.0001 | 0 | 0 | 0 |
| **P54** *MORC2* c.278A>C (p.Glu93Ala) | Neutral (0.59) | Supporting (0.196) | 0 | 0 | 0 | 0 |
| **P55** *MORC2* c.610C>T (p.Leu204Phe) | Hi (3.63) | Uncertain (0.002) | 0 | 0 | 0 | 0 |
| **P56** *MPV17* c.263A>T (p.Lys88Met) | Hi (3.59) | Deleterious (Supporting) (0) | 0.0053 | 0 | 0.0058 | 0 |
| **P56** *MPV17* c.265A>T (p.Met89Leu) | Lo (0.87) | Deleterious (Supporting) (0) | 0.0029 | 0 | 0.0058 | 0 |
| **P57** *MPZ* c.775G>T (p.Gly259Trp) | N/A | N/A | 0.0001 | 0 | 0 | 0 |
| **P58** *MTMR2* c.638del (p.Leu213*) | N/A | N/A | 0 | 0 | 0 | 0 |
| **P59** *MTMR2* c.1164G>A (p.Trp388*) | N/A | N/A | 0 | 0 | 0 | 0 |
| **P60** *NEFH* c.884-1G>T (p.?) | N/A | N/A | 0.0003 | 0 | 0 | 0 |
| **P61** *PIEZO2* c.3544C>T (p.Arg1182Cys) | Med (3.14) | Deleterious (Supporting) (0) | 0.0039 | 0 | 0.0153 | 0 |
| **P62** *PIEZO2* c.104T>C (p.Leu35Pro) | Med (2.07) | Uncertain (0.001) | 0 | 0 | 0 | 0 |
| **P63** *POLG* c.911T>G (p.Leu304Arg) | Med (2.98) | Uncertain (0.001) | 0.0023 | 0 | 0.0075 | 0 |
| **P64** *POLG* c.2243G>C (p.Trp748Ser) | Med (1.96) | Uncertain (0.024) | 0.073 | 0 | 0.0843 | 0 |
| **P65** *PRX* c.2035C>T (p.Arg679*) | N/A | N/A | 0.0006 | 0 | 0 | 0 |
| **P66** *PRX* c.985del (p.Ala329Glnfs*27) | N/A | N/A | 0 | 0 | 0 | 0 |
| **P67** *PRX* c.1390C>T (p.Arg464*) | N/A | N/A | 0.0003 | 0 | 0.0008 | 0 |
| **P68** *RETREG1* c.433C>T (p.Gln145*) | N/A | N/A | 0 | 0 | 0 | 0 |
| **P69** *RNF170* c.304T>C (p.Cys102Arg) | Hi (4.12) | Deleterious (Supporting) (0) | 0.0001 | 0 | 0 | 0 |
| **P70** *SACS* c.8107C>T (p.Arg2703Cys) | Med (3) | Deleterious (Supporting) (0) | 0.0003 | 0 | 0.0008 | 0 |
| **P71** *SACS* c.408_409del (p.Gln137Ilefs*46) | N/A | N/A | 0.0001 | 0 | 0 | 0 |
| **P72** *SACS* c.5347_5348insT (p.Arg1783Metfs*5) | N/A | N/A | 0 | 0 | 0 | 0 |
| **P73** *SACS* c.9508C>T (p.Arg3170*) | N/A | N/A | 0.0008 | 0 | 0.0008 | 0 |
| **P74** *SACS* c.8880dup (p.Ser2961Ilefs*6) | N/A | N/A | 0 | 0 | 0 | 0 |
| **P75** *SACS* c.826C>T (p.Arg276Cys) | Med (3.25) | Deleterious (Supporting) (0) | 0 | 0 | 0.0007 | 0 |
| **P76** *SACS* c.11953G>T (p.Glu3985*) | N/A | N/A | 0 | 0 | 0 | 0 |
| **P77** *SACS* c.5346del (p.Phe1782Leufs*32) | N/A | N/A | 0 | 0 | 0 | 0 |
| **P78** *SACS* c.13288C>T (p.Gln4430*) | N/A | N/A | 0.0001 | 0 | 0 | 0 |
| **P79** *SACS* c.2976_2982del (p.Lys993Phefs*2) | N/A | N/A | 0 | 0 | 0 | 0 |
| **P80** *SACS* c.13288C>T (p.Gln4430Ter) | N/A | N/A | 0.0001 | 0 | 0 | 0 |
| **P81** *SBF2* c.1395+1G>A (p.?) HOM, N | N/A | N/A | 0 | 0 | 0 | 0 |
| **P82** *SCN11A* c.4610_4611del (p.Phe1537Cysfs*31) | N/A | N/A | 0.0009 | 0 | 0.0008 | 0 |
| **P83** *SCN9A* c.4963A>G (p.Met1655Val) | Med (2) | Uncertain (0.021) | 0.0001 | 0 | 0 | 0 |
| **P84** *SETX* c.5243del (p.Phe1748Serfs*3) | N/A | N/A | 0 | 0 | 0 | 0 |
| **P85** *SETX* c.6846G>C (p.Gln2282His) | Neutral (0.28) | Uncertain (0.002) | 0 | 0 | 0 | 0 |
| **P86** *SETX* c.5320C>T (p.Gln1774*) | N/A | N/A | 0.0008 | 0 | 0 | 0 |
| **P87** *SH3TC2* c.856G>C (p.Glu286Gln) | Med (3.2) | Deleterious (Supporting) (0) | 0.0001 | 0 | 0 | 0 |
| **P88** *SH3TC2* c.2860C>T (p.Arg954*) | N/A | N/A | 0.1084 | 0 | 0 | 0 |
| **P89** *SH3TC2* c.1691A>G (p.Asp564Gly) | Med (2.78) | Uncertain (0.022) | 0 | 0 | 0 | 0 |
| **P90** *SH3TC2* c.3839G>A (p.Trp1280*) | N/A | N/A | 0 | 0 | 0 | 0 |
| **P91** *SH3TC2* c.1691A>G (p.Asp564Gly) | Med (2.78) | Uncertain (0.022) | 0 | 0 | 0 | 0 |
| **P91** *NEFH* c.646_653del (p.Leu216Glufs*61) | N/A | N/A | 0 | 0 | 0 | 0 |
| **P92** *SNAP29* c.487dup (p.Ser163Lysfs*6) | N/A | N/A | 0.0016 | 0 | 0.005 | 0 |
| **P93** *SPG11* c.3143_3144dup (p.Asp1049Glnfs*5) | N/A | N/A | 0 | 0 | 0 | 0 |
| **P94** *SPG11* c.250T>C (p.Phe84Leu) | Med (2.22) | Uncertain (0.04) | 0.0014 | 0 | 0.0016 | 0 |
| **P95** *SPG11* c.3075dup (p.Glu1026Argfs*4) | N/A | N/A | 0.0023 | 0 | 0 | 0 |
| **P96** *SPG11* c.4001G>A (p.Arg1334Lys) | Med (2.67) | Uncertain (0.023) | 0 | 0 | 0 | 0 |
| **P97** *SPG11* c.5616G>A (p.Trp1872*) | N/A | N/A | 0 | 0 | 0 | 0 |
| **P98** *SPTBN4* c.1795del (p.Leu599Cysfs*21) | N/A | N/A | 0 | 0 | 0 | 0 |
| **P99** *SPTBN4* c.1789G>A (p.Ala597Thr) | Lo (1.65) | Supporting (0.108) | 0.0031 | 0 | 0.0008 | 0 |
| **P100** *SPTBN4* c.625T>C (p.Trp209Arg) | Hi (4.24) | Deleterious (Supporting) (0) | 0 | 0 | 0 | 0 |
| **P101** *SYNE1* c.18620C>T (p.Thr6207Met) | Lo (1.5) | Uncertain (0.026) | 0.003 | 0 | 0.0058 | 0 |
| **P101** *SYNE1* c.5267G>A (p.Arg1756Lys) | Med (2.72) | Supporting (0.109) | 0.0001 | 0 | 0 | 0 |
| **P102** *SYT2* c.2T>A (p.Met1?) | N/A | Uncertain (0.004) | 0 | 0 | 0 | 0 |
| **P103** *TECPR2* c.911G>A (p.Trp304*) | N/A | N/A | 0 | 0 | 0 | 0 |
| **P104** *TYMP* c.31_32insAACCGGG (p.Ala11Glufs*9) | N/A | N/A | 0 | 0 | 0 | 0 |
| **P105** *TYMP* c.518T>A (p.Met173Lys) | Med (2.86) | Uncertain (0.002) | 0 | 0 | 0 | 0 |
| **P106** *VPS13D* c.2961T>G (p.Phe987Leu) | Med (2.25) | N/A | 0.0009 | 0 | 0.0016 | 0 |
| **P107** *PDXK* c.225T>A (p.Asn75Lys) | Lo (1.19) | Supporting (0.279) | 0 | 0 | 0 | 0 |

MutationAssessor (MUT Assesor) scores are reported as Neutral, Low (Lo), Medium (Med), or High (Hi) functional impact. SIFT predictions are reported together with their corresponding scores; variants predicted to be deleterious are indicated as "Deleterious (Supporting)." "N/A" indicates that no prediction was available for the corresponding variant. Allele frequencies are presented as percentages. Population allele frequencies for each identified variant were extracted from the Genome Aggregation Database (gnomAD), the Exome Aggregation Consortium (ExAC), Iranome, and GenomeAsia databases.
