# Supplementary material for: Expanding the genetic spectrum of inherited peripheral neuropathies in complex neurogenetic disorders using whole-exome sequencing
Source: Neurogenetics. 2026 Oct 29;27(1):85. doi: 10.1007/s10048-026-00952-w (PMC13719752; doi:10.1007/s10048-026-00952-w)
Supplement: Supplementary file 3 [file 10048_2026_952_MOESM3_ESM.docx]

**Supplementary Table 3. Detailed ACMG classification criteria applied to the novel likely pathogenic and pathogenic identified variants.**

| **Patient ID** | **Gene (RefSeq transcript)** | **Nucleotide change (HGVS)** | **Protein change (HGVS)** | **Zygosity** | **ACMG classification** | **ACMG evidence codes** |
| --- | --- | --- | --- | --- | --- | --- |
| P1 | *FLVCR1* (NM_014053.4) | c.1_18del | p.Met1? | HOM | **LP** | PS1 (Strong); PVS1 (Moderate); PM2 (Moderate); PM3 (Supporting) |
| P9 | *ARSA* (NM_000487.6) | c.1283C>G | p.Pro428Arg | HOM | **LP** | PM1 (Moderate); PP2 (Supporting); PM2 (Moderate); PM5 (Moderate); PP3 (Supporting); PM3 (Supporting) |
| P10 | *ARSA* (NM_000487.6) | c.393del | p.Pro132Leufs*16 | HET | **P** | PVS1 (Very Strong); PM2 (Moderate); PM3 (Supporting) |
| P16 | *CNTNAP1* (NM_003632.3) | c.3750C>A | p.Cys1250* | HOM | **LP** | PVS1 (Very Strong); PM2 (Moderate); PM3 (Supporting) |
| P18 | *CYP2U1* (NM_183075.3) | c.491-2A>G | p.? | HOM | **LP** | PVS1 (Strong); PM2 (Moderate); PM3 (Supporting) |
| P21 | *DNAJB2* (NM_006736.6) | c.159T>G | p.Tyr53* | HOM | **P** | PVS1 (Very Strong); PM2 (Moderate); PM3 (Supporting) |
| P22 | *DYNC1H1* (NM_001376.5) | c.10007A>T | p.Lys3336Met | HET | **P** | PS4 (Strong); PM2 (Moderate); PM1 (Supporting); PP2 (Supporting); PM5 (Supporting); PP3 (Supporting) |
| P25 | *FAM126A* (NM_032581.4) | c.229C>T | p.Gln77* | HOM | **P** | PVS1 (Very Strong); PM3 (Strong); PM2 (Moderate) |
| P42 | *MFF* (NM_001277062.2) | c.159del | p.Pro54Argfs*6 | HOM | **P** | PVS1 (Very Strong); PM2 (Moderate); PM3 (Supporting) |
| P43 | *MFF* (NM_001277062.2) | c.352-2A>C | p.? | HOM | **P** | PVS1 (Very Strong); PM2 (Moderate); PM3 (Supporting) |
| P44 | *MFN2* (NM_014874.4) | c.610G>C | p.Asp204His | HET | **P** | PS4 (Strong); PM1 (Moderate); PP2 (Supporting); PM2 (Moderate); PP3 (Supporting) |
| P49 | *MME* (NM_007289.4) | c.2081G>A | p.Trp694* | HOM | **P** | PVS1 (Very Strong); PM2 (Moderate); PS4 (Moderate) |
| P53 | *MME* (NM_007289.4) | c.655-1G>C | p.? | HOM | **P** | PVS1 (Strong); PS4 (Strong); PM2 (Moderate) |
| P58 | *MTMR2* (NM_016156.6) | c.638del | p.Leu213* | HOM | **P** | PVS1 (Very Strong); PM2 (Moderate); PM3 (Supporting) |
| P59 | *MTMR2* (NM_016156.6) | c.1164G>A | p.Trp388* | HOM | **P** | PVS1 (Very Strong); PM3 (Strong); PM2 (Moderate) |
| P60 | *NEFH* (NM_021076.4) | c.884-1G>T | p.? | HET | **LP** | PVS1 (Very Strong); PM2 (Moderate) |
| P66 | *PRX* (NM_181882.3) | c.985del | p.Ala329Glnfs*27 | HOM | **P** | PVS1 (Strong); PS4 (Strong); PM2 (Moderate) |
| P71 | *SACS* (NM_014363.6) | c.408_409del | p.Gln137Ilefs*46 | HOM | **P** | PVS1 (Very Strong); PM2 (Moderate); PM3 (Supporting) |
| P72 | *SACS* (NM_014363.6) | c.5347_5348insT | p.Arg1783Metfs*5 | HOM | **LP** | PVS1 (Strong); PM2 (Moderate); PM3 (Supporting) |
| P74 | *SACS* (NM_014363.6) | c.8880dup | p.Ser2961Ilefs*6 | HOM | **LP** | PVS1 (Strong); PM2 (Moderate); PM3 (Supporting) |
| P76 | *SACS* (NM_014363.6) | c.11953G>T | p.Glu3985* | HOM | **LP** | PVS1 (Strong); PM2 (Moderate); PM3 (Supporting) |
| P77 | *SACS* (NM_014363.6) | c.5346del | p.Phe1782Leufs*32 | HOM | **LP** | PVS1 (Strong); PM2 (Moderate); PM3 (Supporting) |
| P78 | *SACS* (NM_014363.6) | c.13288C>T | p.Gln4430* | HOM | **LP** | PVS1 (Strong); PM2 (Moderate); PM3 (Supporting) |
| P79 | *SACS* (NM_014363.6) | c.2976_2982del | p.Lys993Phefs*2 | HOM | **LP** | PVS1 (Strong); PM2 (Moderate); PM3 (Supporting) |
| P80 | *SACS* (NM_014363.6) | c.13288C>T | p.Gln4430* | HOM | **LP** | PVS1 (Strong); PM2 (Moderate); PM3 (Supporting) |
| P81 | *SBF2* (NM_030962.4) | c.1395+1G>A | p.? | HOM | **LP** | PVS1 (Strong); PM2 (Moderate); PM3 (Supporting) |
| P83 | *SCN9A* (NM_001365536.1) | c.4963A>G | p.Met1655Val | HOM | **LP** | PM2 (Moderate); PP3 (Moderate); PS4 (Moderate) |
| P84 | *SETX* (NM_015046.7) | c.5243del | p.Phe1748Serfs*3 | HOM | **LP** | PVS1 (Very Strong); PM2 (Moderate); PS4 (Moderate) |
| P89 | *SH3TC2* (NM_024577.4) | c.1691A>G | p.Asp564Gly | HOM | **LP** | PM2 (Moderate); PS4 (Moderate); PP3 (Supporting); PM1 (Supporting) |
| P91 | *SH3TC2* (NM_024577.4) | c.1691A>G | p.Asp564Gly | HOM | **LP** | PM2 (Moderate); PS4 (Moderate); PP3 (Supporting); PM1 (Supporting) |
| P91 | *NEFH* (NM_021076.4) | c.646_653del | p.Leu216Glufs*61 | HET | **P** | PVS1 (Very Strong); PM2 (Moderate); PS4 (Moderate) |
| P97 | *SPG11* (NM_025137.4) | c.5616G>A | p.Trp1872* | HOM | **P** | PVS1 (Very Strong); PM2 (Moderate); PM3 (Supporting) |
| P98 | *SPTBN4* (NM_020971.3) | c.1795del | p.Leu599Cysfs*21 | HOM | **P** | PVS1 (Very Strong); PM2 (Moderate); PM3 (Supporting) |
| P103 | *TECPR2* (NM_014844.5) | c.911G>A | p.Trp304* | HOM | **P** | PVS1 (Very Strong); PM2 (Moderate); PM3 (Supporting) |
| P104 | *TYMP* (NM_001953.5) | c.31_32insAACCGGG | p.Ala11Glufs*9 | HOM | **P** | PVS1 (Very Strong); PM2 (Moderate); PM3 (Supporting) |
| P105 | *TYMP* (NM_001953.5) | c.518T>A | p.Met173Lys | HOM | **LP** | PM2 (Moderate); PM5 (Moderate); PP3 (Moderate); PP2 (Supporting); PM3 (Supporting) |

All variants listed were classified as likely pathogenic (LP), pathogenic (P) according to the 2015 American College of Medical Genetics and Genomics Pathology (ACMG) sequence-variant interpretation guidelines [1].

Abbreviations: HOM, homozygous; HET, heterozygous; LP, likely pathogenic; P, pathogenic; ACMG evidence codes: PVS1, null variant in a gene for which loss of function is an established disease mechanism; PS1, same amino acid change as a previously established pathogenic variant; PS4, prevalence of the variant in affected individuals is significantly increased over controls; PM1, located in a mutational hot spot or well-characterized functional domain; PM2, absent (or present at extremely low frequency) in population databases; PM3, detected in trans with a pathogenic variant in a recessive disorder; PM5, novel missense change at an amino acid residue where a different missense change has been established as pathogenic; PP2, missense variant in a gene with a low rate of benign missense variation and in which missense variants are a common mechanism of disease; PP3, multiple computational/in silico algorithms support a deleterious effect on the gene or gene product. The parenthetical term following each evidence code (Very Strong, Strong, Moderate, or Supporting) indicates the evidence strength assigned in the final points-based classification for that variant.

**Reference:**

1.	Richards, S., et al., *Standards and guidelines for the interpretation of sequence variants: a joint consensus recommendation of the American College of Medical Genetics and Genomics and the Association for Molecular Pathology.* Genet Med, 2015. **17**(5): p. 405-24.
